# The prevalence and trends of waterpipe tobacco smoking: A systematic review

**DOI:** 10.1371/journal.pone.0192191

**Published:** 2018-02-09

**Authors:** Mohammed Jawad, Rana Charide, Reem Waziry, Andrea Darzi, Rami A. Ballout, Elie A. Akl

**Affiliations:** 1 Public Health Policy Evaluation Unit, Imperial College London, London, United Kingdom; 2 Clinical Research Institute, American University of Beirut, Beirut, Lebanon; 3 Department of Epidemiology Harvard T.H. Chan School of Public Health, Boston, Massachusetts, United States of America; 4 Faculty of Health Sciences, American University of Beirut, Beirut, Lebanon; 5 AUB GRADE Center, Clinical Research Institute, American University of Beirut, Beirut, Lebanon; 6 Faculty of Medicine, American University of Beirut, Beirut, Lebanon; 7 Department of Internal Medicine, American University of Beirut, Beirut, Lebanon; 8 Department of Health Research Methods, Evidence and Impact, McMaster University, Hamilton, Canada; University College London, UNITED KINGDOM

## Abstract

**Introduction:**

Waterpipe tobacco smoking is harmful to health however its prevalence estimates remain uncertain. We aimed to systematically review the medical literature on waterpipe tobacco prevalence and trends.

**Methods:**

We searched Medline, Embase and ISI Web of Science for ‘waterpipe’ and its synonyms, without using language or date restrictions. We included any measure of waterpipe tobacco smoking prevalence in jurisdictionally representative populations. We stratified findings by prevalence measure (past 30 day, ever, regular or occasional, daily, other or unspecified) and age (adults or youth).

**Results:**

We included 129 studies reporting 355 estimates for 68 countries. In general, prevalence estimates among adults were highest in the Eastern Mediterranean, and among youth were about equal between Eastern Mediterranean and European regions. Past 30 day use was highest among Lebanese youth (37.2% in 2008), ever use was highest among Lebanese youth in 2002 and Lebanese university students in 2005 (both 65.3%), regular or occasional use was highest in among Iranian university students (16.3% in 2005), and daily use was highest among Egyptian youth (10.4% in 2005). Trend data were limited but most studies reported increased use over time, ranging from 0.3–1.0% per year among youth in the US to 2.9% per year among youth in Jordan (both for past 30 day use). Results were similar for ever use trends. Turkey (2.3% in 2008 to 0.8% in 2010) and Iraq (6.3% in 2008 and 4.8% in 2012) both witnessed decreased waterpipe use.

**Conclusion:**

Waterpipe tobacco smoking is most prevalent in Eastern Mediterranean and European countries, and appears higher among youth than adults. Continued surveillance will be important to assess and inform policy measures to control waterpipe tobacco use.

## Introduction

Waterpipe tobacco smoking involves the use of a multi-stemmed instrument containing water at its base through which tobacco smoke, often fruit-flavoured, passes prior to inhalation. Several systematic reviews and meta-analyses have shown this method of tobacco consumption to be linked to diseases typically associated with cigarette use, such as lung cancer, oral cancer, cardiovascular disease, respiratory disease, and low birth weight[1, 2]. Despite this, both waterpipe tobacco users and non-users consider waterpipe tobacco to be less harmful than cigarette tobacco[3]. Reasons for such perceptions include the socialisation, relaxation, pleasure and entertainment that waterpipe tobacco use brings to the user[4]. Moreover, effective policies addressing waterpipe tobacco use in settings of highest burden are lacking[5].

The World Health Organization Global Action Plan has set a target of a 25% relative reduction in tobacco use by 2025[6]. However, evidence suggests that waterpipe tobacco smoking may undermine this target given its anecdotal increase in prevalence across multiple settings. A systematic review conducted in 2008 showed that waterpipe tobacco smoking prevalence was alarmingly high among school and university students within Middle Eastern countries as well as those of Middle Eastern descent within Western countries[7]. However, only four studies in this review were nationally representative, so the epidemiological picture of waterpipe tobacco use remains uncertain.

The research profile of waterpipe tobacco smoking has increased in recent years: it was a central theme at the 16^th^ World Conference on Tobacco or Health held in Abu Dhabi in 2015[8] and at the subsequent Seventh Conference of the Parties held in India in 2016[9]. The World Health Organization has also recently published an updated advisory note outlining the latest literature[10], and a special journal supplement has been dedicated to waterpipe tobacco smoking research[11]. While these show an exponentially rising number of prevalence studies, references to these often unsystematic and focus on a selective number studies in a limited number of countries and typically at non-jurisdictionally representative level (e.g., single centre studies among college students)[12, 13]. These approaches are likely to introduce bias that could under- or over-estimate the prevalence, and therefore importance, of waterpipe tobacco use in the discourse, around policy measures. This is particularly important to address within waterpipe tobacco research given a relatively high prevalence among college students compared with other population groups[7]. Therefore, this study aimed to systematically review the medical literature on the prevalence and trends of waterpipe tobacco smoking in jurisdictionally representative populations.

## Methods

### Eligibility criteria

Our inclusion criteria were:

- Study design: cross-sectional studies (for prevalence) and cohort studies (for incidence), with probability or census sampling;
- Population: among the general population or demographically defined populations (e.g. defined by age, sex, or ethnicity), including populations within educational establishments, at the level of a jurisdiction (as defined by the authors);
- Outcome: any measure of waterpipe tobacco smoking prevalence.

Our exclusion criteria were:

- Study design: case control studies, case reports, case series, interventional studies, studies not reporting original research
- Representativeness: studies using non-probability sampling or, if using probability sampling, not sufficiently covering the jurisdiction (e.g. studies conducted in single institutions)
- Population: occupationally defined populations (e.g. among doctors only) or other non-demographic features (e.g. among non-cigarette smokers or among those with a certain hobby);
- Outcome: waterpipe smoking use for non-tobacco products (e.g. cannabis, opium); waterpipe tobacco smoking prevalence not provided separately from data on other forms of tobacco use.

We also excluded studies reported as abstracts for which we could not identify a full text after consultation of a medical librarian or contact with the corresponding author.

### Search strategy

We conducted our initial search in February 2016. Then we put in place a literature surveillance process to find additional studies throughout the development of this review, which ended in January 2017. We searched Medline, Embase and ISI Web of Science with no language or date restrictions. We used “waterpipe” along with its synonyms and their spelling variations, e.g., “hookah”, “shisha”, “narghile” and other culturally-specific terms (Table A in S1 File). Two medical librarians reviewed and advised on the search strategy.

In addition, we hand-searched the reference lists of included studies and review articles and used the Google Scholar function to find articles that cited relevant studies. We requested additional unpublished data from the corresponding authors of studies which failed to distinguish between waterpipe tobacco smoking and other forms of tobacco use. We also contacted the corresponding authors of studies for which we could not acquire a full text.

### Selection process

Two teams of reviewers (MJ/RW and RB/RW) screened in duplicate and independently the titles and abstracts of captured citations to identify potentially eligible studies. We retrieved full texts of studies considered potentially eligible by at least one reviewer, taking a more inclusive approach to abstracts that appeared somewhat suitable for inclusion. We conducted a calibration exercise to identify common areas of discordance and the need to better clarify the eligibility criteria; the inter-rater reliability was not calculated. Three teams of reviewers (MJ/RC, MJ//RB/RW, MJ/AD) screened, in duplicate and independently the full texts using a standardised and pilot-tested screening form. They resolved disagreements by discussion, and when needed, with the help of a third reviewer (EAA).

### Data abstraction

Three teams of reviewers (MJ/RC, MJ/RW, MJ/AD) conducted a calibration exercise before abstracting data from each eligible study in duplicate and independently using a standardised and pilot-tested data abstraction form. They resolved disagreements by discussion, and when needed with the help of a third reviewer (EAA). We abstracted data in the following areas:

- Governance: funding source, ethics approval, and conflicts of interest;
- Design features: sampling frame, sampling method, survey recruitment method, survey administration method
- Methodological quality: sample size calculation, validity of survey tool, pilot testing, response rate
- Population age and setting: mean age or age range, percentage of males, country, level of jurisdiction (national, subnational, city), study timing, and the numbers sampled, participated, and analysed.
- Results: We reported the prevalence measure, taken verbatim from the study, in addition to the prevalence estimate

### Quality assessment

We quantitatively rated the quality of studies using a modified version of the Newcastle-Ottawa Scale for cross-sectional studies[14]. The ‘selection’ domain, out of five, included the representativeness of the sample, sample size, non-respondents, and ascertainment of the exposure. Given our inclusion criteria restricted this review to jurisdictionally-representative samples, all included studies would score least one point on the ‘selection’ domain. The ‘outcome’ domain, out of three, included the assessment of the outcome and the statistical test. We scored studies one point for the statistical test where they provided the minimum information required to construct 95% confidence intervals (95% CIs) around their prevalence estimates (see below for our calculation). We did not rate studies on the ‘comparability’ domain as this was not relevant to our research question.

### Data analysis

We categorised countries by WHO region (Africa, Americas, Eastern Mediterranean, Europe, South East Asia, Western Pacific), and population age into adults or youth, with the latter defined as over 50% of participants being aged less than or equal to 24, or the mean age being less than or equal to 24. The definition of ‘youth’ was adapted from the United Nations definition[15]. We recoded study timing to correspond to the first year of data collection; if no data collection year was provided, we used the year prior to the date of publication. We merged ‘city’ and ‘subnational’ into one category due to low numbers. We also categorised verbatim prevalence measures into six: past 30 day use, ever use, regular or occasional use, daily use, other defined use, and unspecified use. We focused our review on the former four measures. The categorisation process can be found in Table B in S1 File.

For each prevalence estimate, and when needed information was reported, we manually constructed 95% CIs[16] and used these even in cases where 95% CIs were reported by the authors.

To ascertain the impact of the quality of studies on waterpipe prevalence, we constructed a linear regression model of all included studies with prevalence as the outcome and with study features/quality as independent variables. The independent variables included the presence of sample size calculation, validity of survey tool, presence of pilot testing, the Newcastle-Ottawa Scale (divided into the selection and outcome domains), and the jurisdiction and location of the study. This regression model was additional adjusted for potential confounders: year of data collection, survey tool used, population age, country of study, and prevalence measure.

We reported estimates stratified by prevalence measure (past 30 days, ever, regular or occasional, daily, other or unspecified), dividing each prevalence measure analysis into an adult analysis, youth analysis, and trend analysis, if applicable. In cases of more than five studies per prevalence measure per region per population age, we reported country-weighted regional mean estimates, with individual studies reported in the Tables C-H in S1 File. For survey tools with a large number of countries, we presented these graphically.

## Results

### Description of included studies

**Fig 1:**
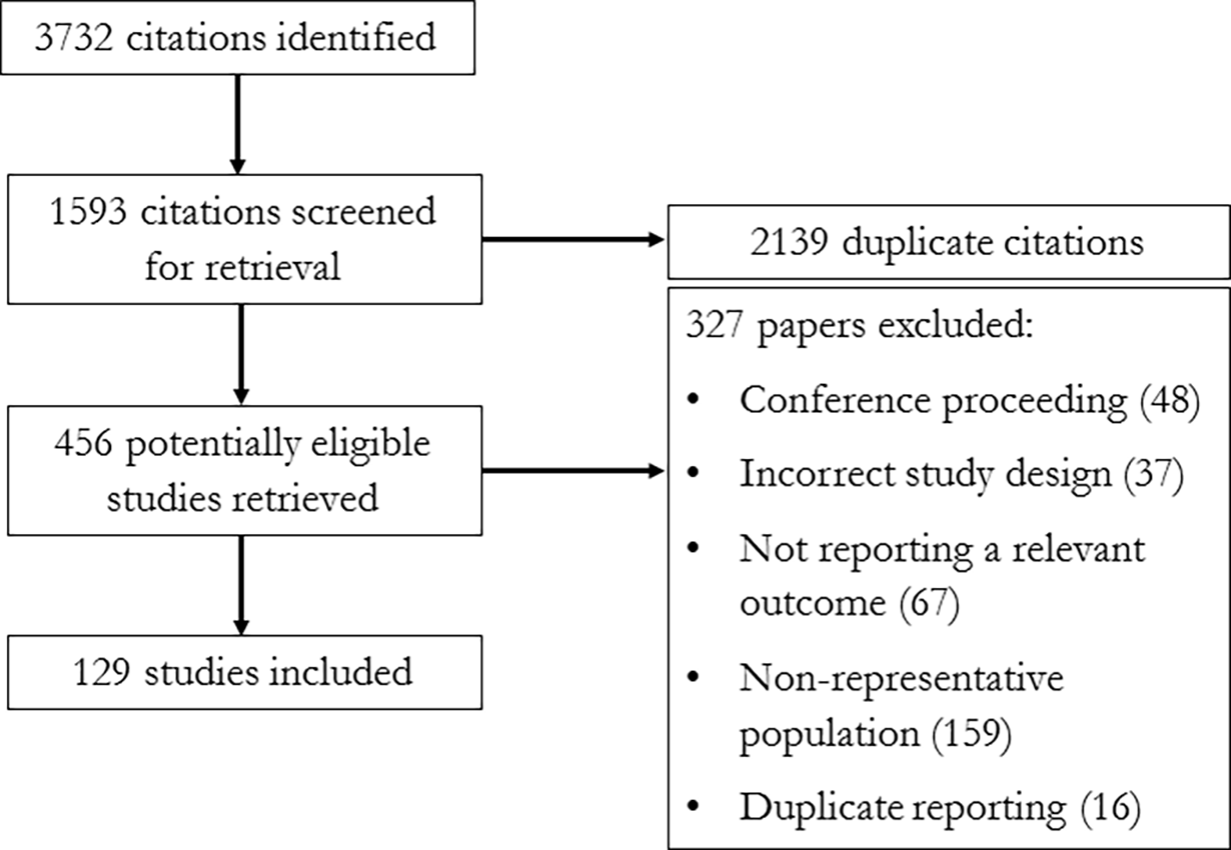
Study flow.

Fig 1 shows the study flow. We considered 456 full texts and excluded 327 for the following reasons: conference proceeding (n = 48), incorrect study design (n = 37), not reporting a relevant outcome (n = 67), non-representative population (n = 159), and duplicate reporting (n = 16).

We included 129 studies, which reported 355 prevalence estimates for waterpipe tobacco use across 68 countries. No studies reported on waterpipe tobacco incidence. Table 1 summarizes the characteristics associated with these prevalence estimates including our assessment of methodological quality. In our linear regression model, studies scoring higher on the Newcastle-Ottawa Scale domain ‘selection’ had lower prevalence estimates, but this was not significant (beta coefficient (B) -2.52, 95% CI -5.2, 0.16, p = 0.065). There was no association between prevalence estimates and the Newcastle-Ottawa Scale domain ‘outcome’, presence of a sample size calculation, validity of survey tool used, presence of pilot testing, jurisdictional level of the study, and location of the study (Table 2), suggesting an unlikelihood that study design features upwardly or downwardly biased our abstracted prevalence estimates (Table 2).

**Table 1:** Characteristics associated with prevalence estimates (N = 355).

Characteristic		% (n)
**Methodological quality**		
Sample size calculation	Reported	*49*.*6 (175)*
Validity of survey tool	Previously developed tool, no validation reported	*25*.*8 (91)*
	Previously developed tool, validation reported	*31*.*4 (111)*
	Self-developed tool, no validation reported	*0*.*6 (2)*
	Self-developed tool, validation reported	*3*.*1 (11)*
	Not reported or unclear	*39*.*1 (138)*
Pilot testing	Reported	*41*.*6 (147)*
Response rate	Reported	*57*.*5 (203)*
Newcastle-Ottawa Scale	Selection domain	*3*.*0 (1*.*2)**^*
	Outcome domain	*2*.*0 (0*.*1)**^*
**Population and setting**		
World Region	Eastern Mediterranean	*36*.*8 (130)*
	Americas	*34*.*8 (123)*
	Europe	*21*.*0 (74)*
	Pacific	*3*.*1 (11)*
	South East Asia	*2*.*8 (10)*
	Africa	*1*.*4 (5)*
Country	USA	*29*.*2 (103)*
	Iran	*10*.*2 (36)*
	Lebanon	*5*.*7 (20)*
	Jordan	*5*.*1 (18)*
	Others	*49*.*9 (176)*
Jurisdiction	National	*68*.*6 (242)*
	Subnational	*31*.*4 (111)*
Population age	Youth	*43*.*6 (152)*
	Adults	*56*.4 (199)*
First year of data collection	2001 to 2005	*8*.*8 (31)*
	2006 to 2010	*49*.*0 (173)*
	2010 to 2015	*42*.*2 (149)*
Survey tool	Global Adult Tobacco Survey	*11*.*6 (41)*
	Eurobarometer Survey	*7*.*1 (25)*
	Global Youth Tobacco Survey	*16*.*1 (57)*
	Other	*39*.*9 (141)*
	Not reported	*25*.*2 (89)*
Prevalence measure	Past-30 day	*30*.*6 (108)*
	Ever	*26*.*3 (93)*
	Regular or occasional	*21*.*0 (74)*
	Daily	*3*.*4 (12)*
	Other	*13*.*9 (49)*
	Unspecified	*4*.*8 (17)*

^Mean (standard deviation)- ‘selection’ domain out of five, ‘outcome’ domain out of three

*adds to 351 as estimates from two studies did not provide adequate information to classify participants as either youth or adults.

**Table 2:** The association between study design features and prevalence estimates.

Characteristic		Beta coefficient (95% CI)	p-value
Sample size calculation	Not reported	*0*.*00*	
	Reported	*0*.*18 (-4*.*27*, *4*.*64)*	*0*.*937*
Validity of survey tool	Not reported or unclear	*0*.*00*	
	Previously developed tool, no validation reported	*4*.*39 (-1*.*73*, *10*.*51)*	*0*.*159*
	Previously developed tool, validation reported	*4*.*71 (-1*.*77*, *11*.*19)*	*0*.*154*
	Self-developed tool, no validation reported	*-7*.*15 (-21*.*98*, *7*.*68)*	*0*.*344*
	Self-developed tool, validation reported	*-0*.*65 (-11*.*46*, *10*.*17)*	*0*.*906*
Pilot testing	Not reported	*0*.*00*	
	Reported	*-1*.*91 (-6*.*00*, *2*.*17)*	*0*.*357*
Newcastle-Ottawa Scale*	Selection domain	*-2*.*52 (-0*.*18*, *0*.*84)*	*0*.*065*
	Outcome domain	*1*.*77 (-7*.*28*, *10*.*83)*	*0*.*700*
Jurisdiction	National	*0*.*00*	
	Subnational	*1*.*30 (-2*.*52*, *5*.*13)*	*0*.*503*
	City	*4*.*73 (-0*.*08*, *9*.*53)*	*0*.*054*
Location	Household	*0*.*00*	
	Hospital/Clinic	*5*.*05 (-1*.*42*, *11*.*53)*	*0*.*126*
	College/University	*6*.*58 (-1*.*52*, *14*.*69)*	*0*.*111*
	School	*3*.*24 (-2*.*93*, *9*.*41)*	*0*.*303*

**Note**: Model additionally adjusted for year of data collection, survey tool used, population age (adults/youth), country, and prevalence measure (past 30 day use, ever use, regular/occasional use, daily use, other use, unspecified). Omitted variables due to collinearity: response rate, WHO region

*Continuous variable.

### Regional estimates

Table 3 presents country-weighted regional mean prevalence estimates by measure and by population age, where more than five studies were available per stratification. In general, prevalence estimates among adults were highest in the Eastern Mediterranean, and among youth were about equal between Eastern Mediterranean and Europe regions. For example, regular or occasional use among adults was 7.2% in the Eastern Mediterranean, 3.8% in the Americas, and less than 1% in South East Asia, the Americas, and the Western Pacific. Youth estimates were only available for three regions and past 30 day use, for example, was highest in Europe (10.6%), then the Eastern Mediterranean (10.3%) and the Americas (6.8%). There was a notable lack of comparable data from Africa, South East Asia, and the Western Pacific.

**Table 3:** Country-weighted regional mean prevalence estimates by prevalence measure and by population age (n: Number of studies).

Region	Adults				Youth			
	Past 30 day	Ever	Regular or occasional	Daily	Past 30 day	Ever	Regular or occasional	Daily
Africa	- (n = 1)	- (n = 1)	- (n = 1)	- (n = 1)	- (n = 0)	- (n = 0)	- (n = 0)	- (n = 0)
Americas	2.0 (n = 9)	9.6 (n = 17)	0.4 (n = 9)	- (n = 0)	6.8 (n = 41)	18.3 (n = 26)	- (n = 0)	- (n = 3)
South East Asia	- (n = 1)	- (n = 0)	0.7 (n = 7)	- (n = 0)	- (n = 0)	- (n = 0)	- (n = 0)	- (n = 0)
Europe	- (n = 0)	- (n = 2)	3.8 (n = 36)	- (n = 0)	10.6 (n = 12)	31.8 (n = 11)	- (n = 1)	- (n = 1)
Eastern Mediterranean	- (n = 4)	31.9 (n = 6)	7.2 (n = 8)	3.2 (n = 5)	10.3 (n = 39)	28.6 (n = 29)	- (n = 0)	- (n = 1)
Western Pacific	- (n = 0)	- (n = 0)	0.7 (n = 8)	- (n = 1)	- (n = 1)	- (n = 0)	- (n = 0)	- (n = 0)

### Past 30 day prevalence

Fourteen studies from six countries (India, Iran, Jordan, Lebanon, South Africa, and the US) reported on past 30 day use among adults[17–30]. National and subnational household surveys from the US conducted between 2009 and 2012, ranged from 0.3% to 3.0% prevalence, with studies restricted to younger adults displaying higher prevalence estimates[17–23]. One US study reported particularly high prevalence, among university students, at 7.8%[30]. Other countries had higher prevalence estimates than the US: past 30 day use was reported as 2.7% in a subnational household survey among women in India in 2014[24], 6.3% among women and 11.0% among both sexes in two city-wide household surveys in Iran conducted in 2010[25] and 2012[27] respectively, 8.7% among pregnant women in maternity clinics in Jordan in 2011[28], 9.9% among university students in a subnational survey in South Africa in 2013[26], and 32.3% among university students in Lebanon in 2005[29].

**Fig 2:**
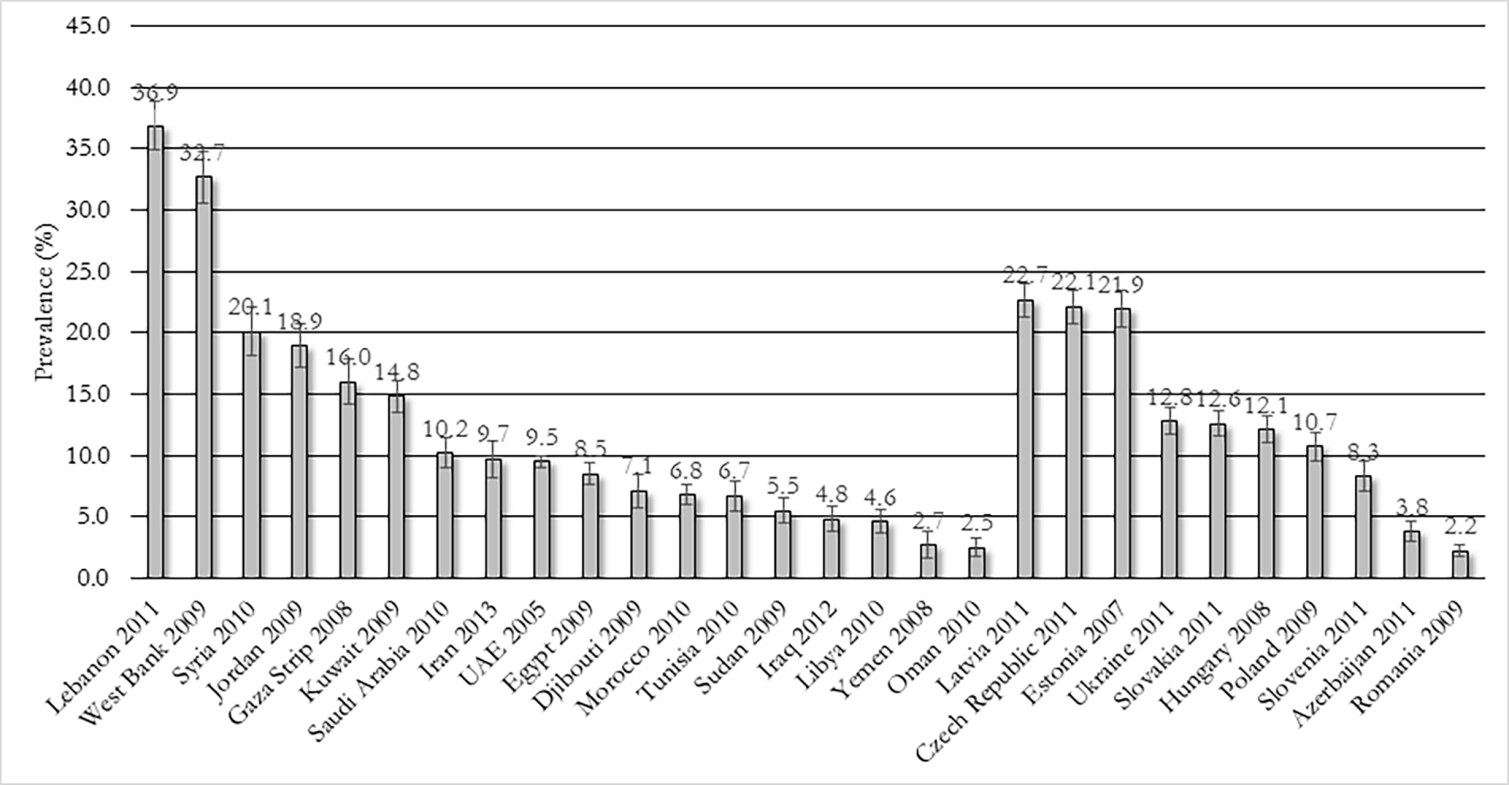
Past 30 day waterpipe tobacco use among youth in the Eastern Mediterranean (left) and European (right) Regions, latest Global Youth Tobacco Survey waves (error bars denote 95% CIs).

Forty four studies from 32 countries reported on past 30 day use among youth[31–75]. Data from these studies, stratified by WHO region, are presented in Table C in S1 File. In general, the Eastern Mediterranean Region had the highest prevalence estimates ranging from 2.5% in Oman to 37.2% in Lebanon. The European Region has slightly lower prevalence estimates, ranging from 2.2% in Romania to 22.7% in Latvia. The Region of the Americas had among the lowest estimates, ranging from 1.0% to 11.4% in the US. Studies reporting on the GYTS were limited to countries in the Eastern Mediterranean and European Regions; their latest waves are presented in Fig 2[37, 66–68], showing that the Czech Republic, Estonia, Latvia, Lebanon, Syria, and the West Bank all report greater than 20% past 30 day prevalence.

### Past 30 day trends

**Fig 3:**
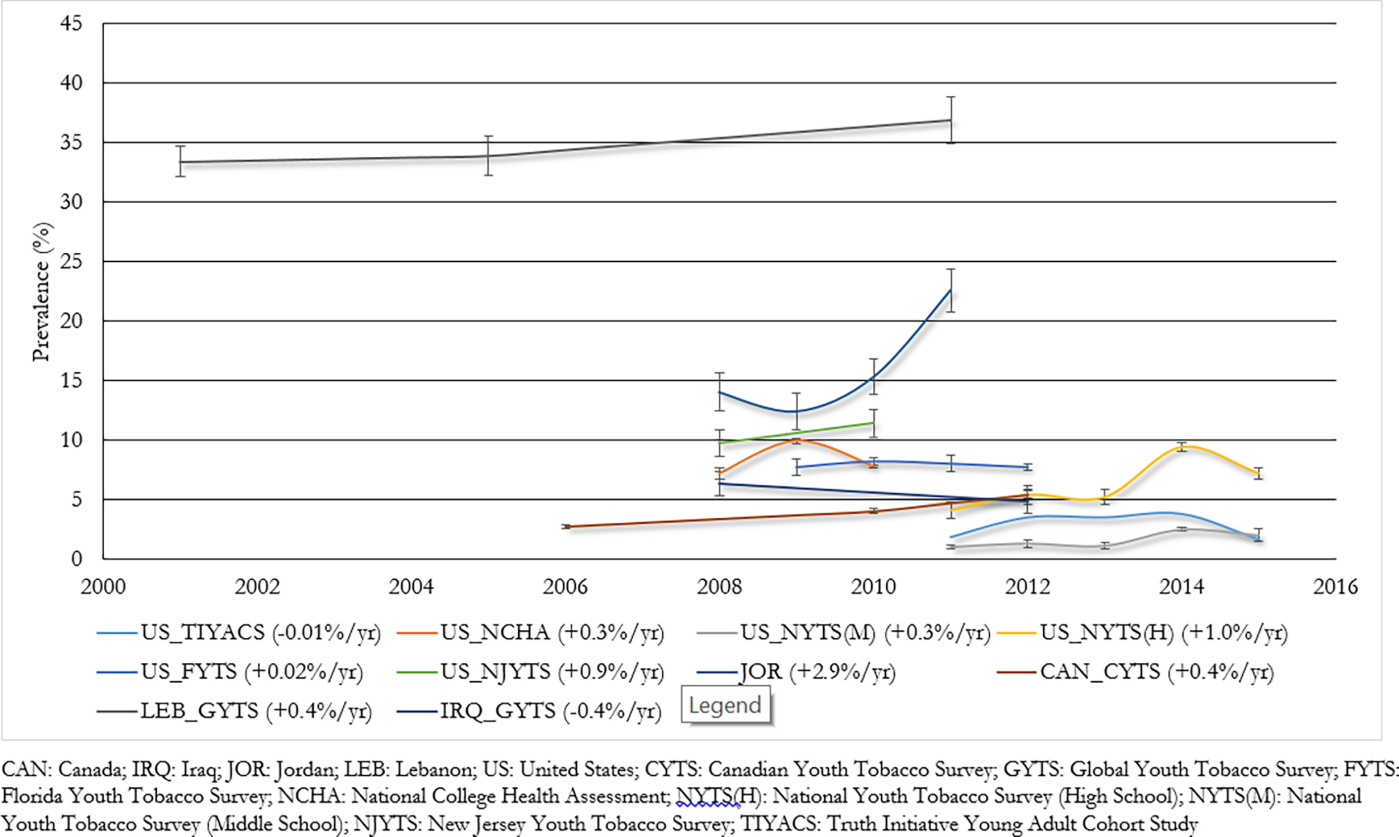
Trends in past 30 day waterpipe use (error bars denote 95% CIs).

Fig 3 shows trends for past 30 day waterpipe tobacco use. Two cross-sectional studies from Iraq showed a 0.4% annual decrease in waterpipe prevalence[37, 52], while all other studies showed either increased or stable trends. In the US, stable trends were observed in Florida (at around the 8% mark between 2009 and 2012)[47] and in the national Truth Initiative Young Adult Cohort Study (at around the 2–3% mark between 2011 and 2016)[32]. In US studies that reported increased trends, this ranged between 0.3% and 1.0% each year[30, 31, 33, 46, 48–51]. In national school-based surveys in Canada, Jordan, and Lebanon, annual increases in prevalence were estimated at 0.4%, 0.4% and 2.9%, respectively[55, 57, 66, 70, 73].

### Ever prevalence

Twenty studies from eight countries (Iran, Israel, Jordan, Lebanon, South Africa, Syria, the UK, and the US) reported on ever use among adults[17–20, 26–29, 76–87]. Data from these studies, stratified by WHO region, are presented in Table D in S1 File. One study from the African Region reported 63.0% ever use in a subnational study among university students in South Africa in 2013[26]. This was one the highest estimates in the review. As per past 30 day estimates, the Eastern Mediterranean Region, reported the highest prevalence of ever use, ranging from 15.8% in Aleppo (Syria), in 2004, to 65.3% among university students in Lebanon[27–29, 77, 78, 81]. Studies from the Region of the Americas and Europe reported much lower prevalence estimates: 11 studies from the US ranged from 1.9% to 21.8%[17–20, 79, 80, 83–87], while in Europe, ever use was reported as 11.6% among adults in the UK in 2012[82], and 19.1% among adults in a subnational household survey in Israel in 2012[76].

Fifty studies from 21 countries reported on ever use among youth[41–75, 88–107]. Data from these studies, stratified by WHO region, are presented in Table E in S1 File. Again, the Eastern Mediterranean Region reported the highest estimates, ranging from 12.9% among secondary school students in Baghdad (Iraq) in 2008 to 65.9% among secondary school students in Beirut (Lebanon) in 2002. In the European Region, prevalence estimates ranged from 12.0% among secondary school students in an English city to 49.5% among secondary school students in Sweden in 2011. In the Region of the Americas, prevalence estimates ranged from 3.0% among secondary school students to 44.0% among adolescents and young adults in the US.

### Ever use trends

**Fig 4:**
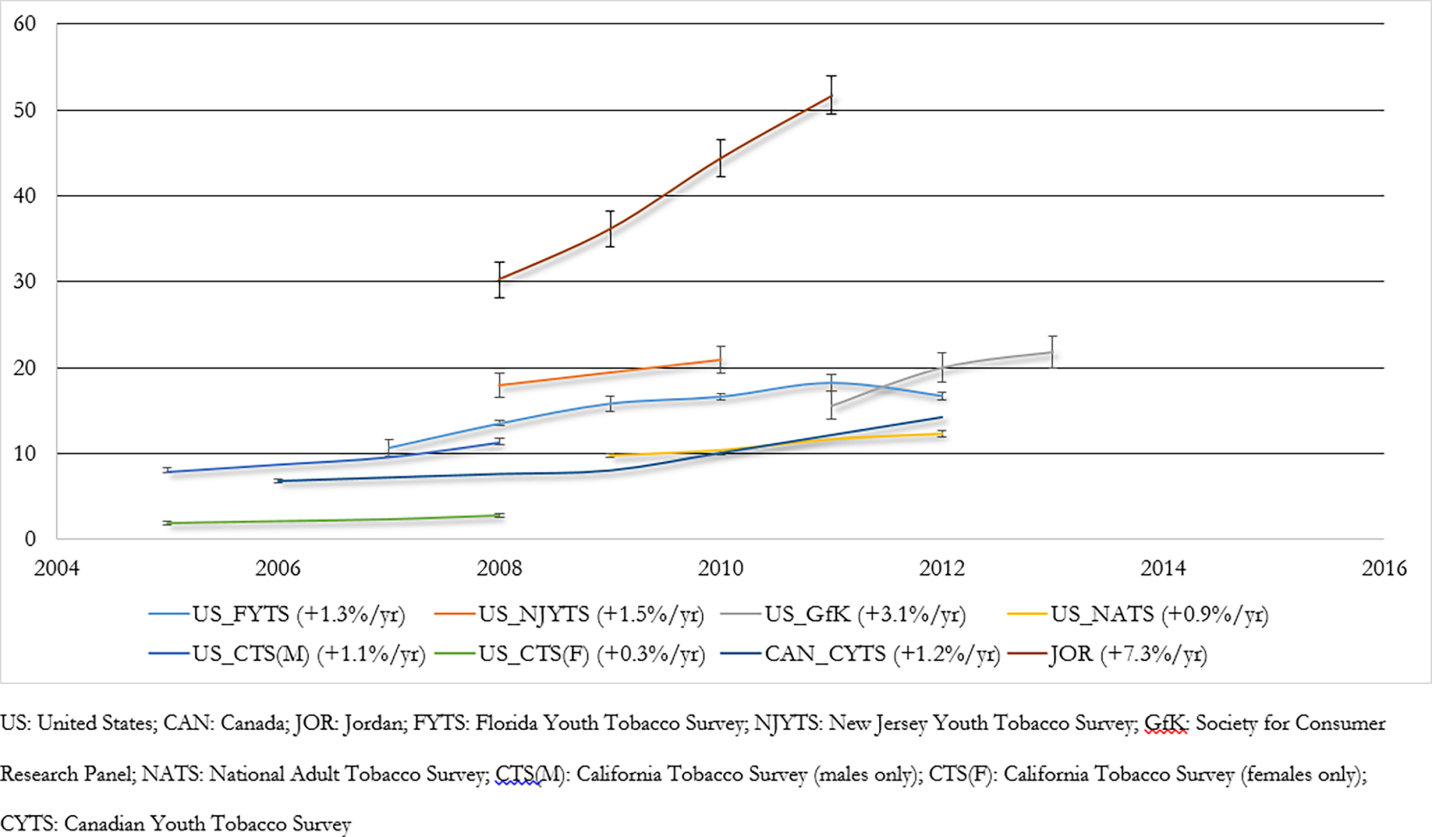
Trends in ever waterpipe use (error bars denote 95% CIs).

Fig 4 shows trends for ever waterpipe tobacco use. Various national and subnational surveys from the US documented absolute increases of between 0.3% and 3.1% per year[20, 47, 49, 85–87]. Repeated rounds of a national school survey in Canada showed an absolute increase of 1.2% per year[55–57], and a similarly designed subnational study in Jordan reported an absolute increase of 7.3% per year between 2008 and 2011[70].

### Regular or occasional use

**Fig 5:**
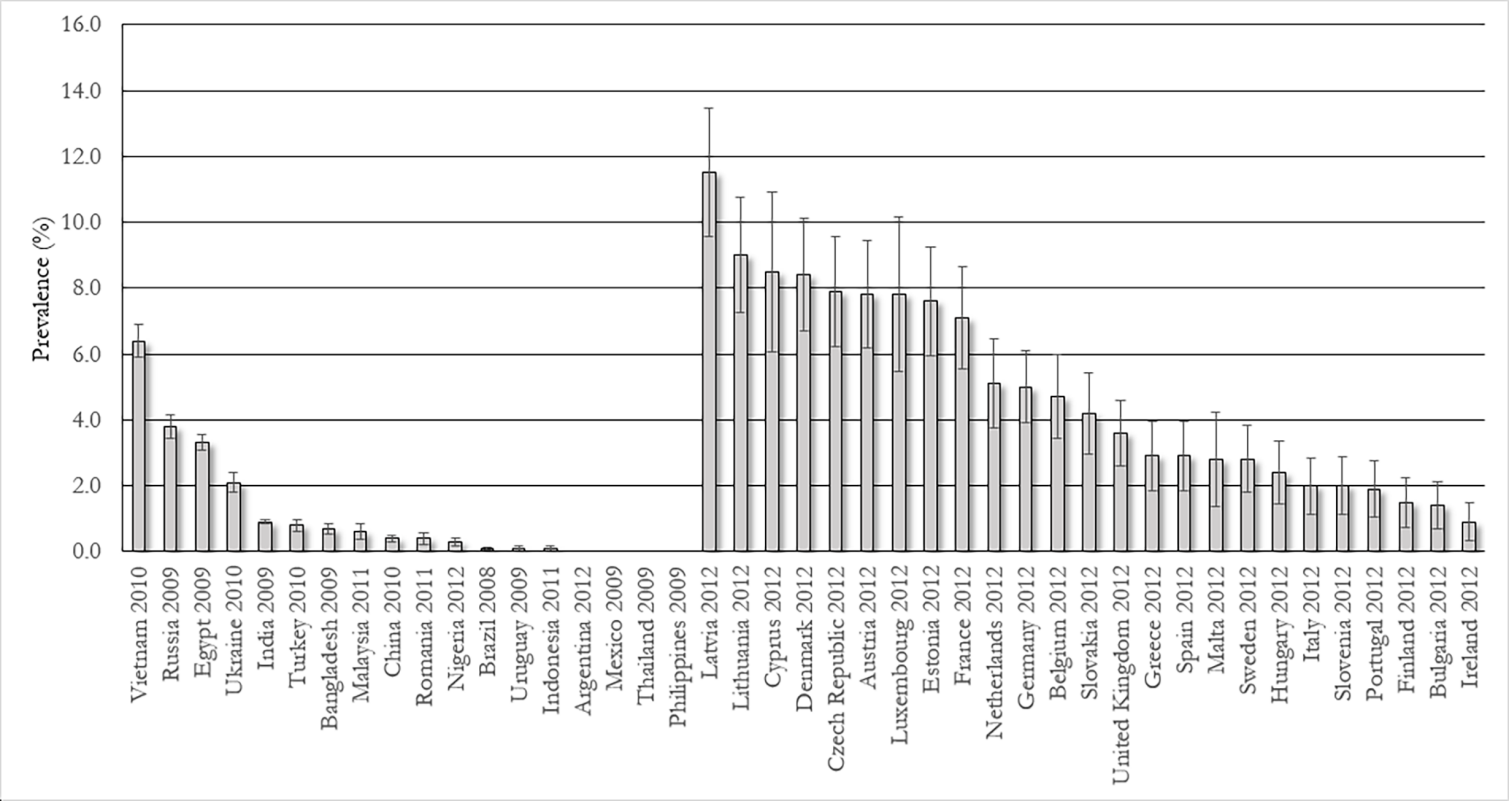
Regular or occasional waterpipe tobacco use among adults, according to the Global Adult Tobacco Survey (left) and Eurobarometer Survey (right) (error bars denote 95% CIs).

Fifteen studies from 48 countries reported regular or occasional use among adults[34, 76–80, 108–116]. Data from these studies, stratified by WHO region, are presented in Table F in S1 File. Once more the Eastern Mediterranean Region reported the highest estimates ranging from 3.3% in Egypt to 16.3% in Iran[77, 78, 108–110]. The European Region was the next most highly prevalent region, with estimates ranging from 0.4% in Romania to 12.8% in Israel[76, 110, 113]. In the South East Asia and Western Pacific Regions they ranged from 0% in Thailand and the Philippines to 6.4% in Vietnam[110]. In the Region of the Americas, prevalence estimates ranged from 0% in Argentina and Mexico, to 1.2% in Brazil[79, 80, 110–112]. Fig 5 summarises these data, and shows the latest wave of the GATS and Eurobarometer surveys, which together include 44 countries.

Only one study measured regular or occasional use among youth. This was a school-based study in London (United Kingdom) and reported a prevalence of 7.6%[99]. Furthermore, only one study measured trends in regular or occasional use. This was using the Turkey GATS data, and it reported a decrease from 2.3% to 0.8% on the 2008 and 2010 surveys[113].

### Daily use

Seven studies from five countries reported daily waterpipe tobacco use among adults[27, 78, 108, 116–119]. Five were from the Eastern Mediterranean Region, and reported prevalence estimates between 1.4% in Syria in 2004 and 4.3% in Saudi Arabia in 2013[27, 78, 108, 118, 119]. A national study from Uganda reported the lowest daily prevalence, at 0.1% in 2014[117], whereas the Vietnam GATS reported the highest daily prevalence at 5.4% in 2010[116].

Five studies from three countries reported daily waterpipe tobacco use among youth[50, 71, 90, 95, 120]. Four of these, one from Estonia in 2006 and three from the US between 2008 and 2013, reported less than 1% prevalence[50, 71, 95, 120]. One subnational study from Egypt reported 10.4% daily waterpipe use[90].

### Other and unspecified use

Thirty studies from 19 countries reported on ‘other’ defined prevalence measures[35, 69, 79, 81, 82, 85, 91–98, 108, 112, 120–132], the details of which are presented in Table G in S1 File. Sixteen studies from ten countries did not specific the waterpipe prevalence measure[88, 123, 129, 133–145], the details of which are presented in Table H in S1 File.

## Discussion

Representative estimates of waterpipe tobacco use are now available in 68 countries across all continents. Most of the included surveys were nationally representative, conducted within the last 10 years, and evenly split between adult and youth populations. While our review shows unsurprisingly high (often >10%) prevalence estimates in the Eastern Mediterranean region of current waterpipe use, particularly the Levant region and among youth, it unexpectedly shows similarly high estimates in several European countries. Except for two US surveys and one from Iraq, all other trend estimates suggest increasing prevalence, regardless of the prevalence measure used.

Our study has a number of strengths. First, we have conducted it following standard systematic review methodology[146], and reported it following the PRISMA guidelines (S2 File). Second, we included only surveys that used random or census sampling to construct representative samples. This helped focus our review only on the better quality studies. Limitations of our review relate to those of the existing literature. For example, we were unable to quantitatively synthesise data by country given the lack of standardisation in survey tools and populations under assessment. We have tried to mitigate this by presenting country-weighted mean regional estimates and by presenting separately data for three main survey tools (GATS, GYTS, and Eurobarometer), which together make up over a third of all of our estimates. These provide a good indication of the between-country differences in waterpipe use. Another limitation is the lack of information regarding cigarette prevalence in these settings, and the extent of dual and poly-tobacco use, however we hope to address this in future reviews. Data from many adult surveys, particularly GATS, report a low prevalence of waterpipe tobacco use which on the face of it creates an impression that waterpipe tobacco is of low public health importance. However, in 13 countries from GATS/Eurobarometer surveys (Argentina, Brazil, China, India, Indonesia, Ireland, Malaysia, Mexico, Nigeria, the Philippines, Romania, Thailand, and Uruguay) with <1% prevalence estimates among adults, only two (Brazil and Romania) had any youth waterpipe prevalence data: both Brazil (9.1%) and Romania (2.2%) reported past-30 day estimates among youth that would warrant interventional attention. Therefore, we advise caution around policymaking decisions resulting from single estimates of adult data and from one survey tool, and call for researchers to triangulate prevalence estimates to build a more informative epidemiological picture.

Variation in survey tools remains problematic. While over half our estimates used ‘ever’ and ‘past 30 day’ indicators, which are arguably quite consistent, about 20% of our estimates (including those from the GATS and Eurobarometer surveys) used a hybrid of ‘occasional or regular’ indicators, which are less consistent (Table B in S1 File). For example, Eurobarometer measures ‘regular or occasional’ among all survey respondents whereas GATS measures ‘daily or non-daily’ use only among those self-identifying as a tobacco user (gateway question: “Do you currently smoke tobacco on a daily basis, less than daily, or not at all?”). The extent to which these are comparable measures remains uncertain, and may partly explain why countries such as Egypt (GATS prevalence 3.3%), where waterpipe tobacco is considered endemic, report low prevalence in comparison to Europe (mean prevalence: 4.6%).

Our review proposes several research implications. Despite the fivefold increase in representative waterpipe tobacco prevalence studies between 2001–2005 and 2006–2010, important gaps remain. Those gaps are more evident when prevalence estimates are stratified by population age (adult vs. youth) and prevalence measure (ever, past 30 day, etc) (Table 3). Two major gaps are the lack of defined ‘current’ adult data from the Eastern Mediterranean region, where waterpipe tobacco is considered endemic, as well as the lack of youth data where GATS is employed e.g. in European, South East Asia, and Western Pacific Regions. Researchers should consider the use of standardised tools so that between-country comparisons can be made[147]. Our review has shown there is a need for interventions to address waterpipe tobacco use. Unfortunately, a recent systematic review suggested a lack of evidence for the effectiveness of most waterpipe interventions[148], so these need to developed and tested for short and long term impact.

The review has also implications for health policy. Given the harm profile of waterpipe tobacco, it would be prudent for countries experiencing high prevalence to make on par waterpipe and cigarette tobacco policy. Globally, a number of potential loopholes exempt waterpipe tobacco from strong, behaviour-changing policy measures such as smoke-free laws and health warning labelling[5]. Calls have been made for a waterpipe-specific policy framework to account for this, which include stronger regulatory action towards waterpipe tobacco paraphernalia and accessories, in addition to a unique regulatory approach to commercial waterpipe tobacco premises[149]. Examples include the application of health warning labels to the waterpipe apparatus itself, the development of zoning laws which only permit commercial waterpipe tobacco premises to operate away from educational and/or residential establishments, and a unique taxation structure that covers the non-tobacco components of waterpipe smoking[5].

To conclude, representative waterpipe tobacco prevalence estimates are now available in 68 countries. Prevalence estimates among adults are highest in the Eastern Mediterranean region, though among youth they are substantially higher across measures; the highest estimates are found in both Eastern Mediterranean and European regions. Continued surveillance is warranted to better inform an epidemiological picture of waterpipe tobacco use, and policy measures should be strengthened to address waterpipe.

## Supporting information

S1 File Search strategies, prevalence definitions, and data from individual studies. (DOCX)

S2 File PRISMA checklist. (DOC)
