# Supplementary material for: The prevalence and trends of waterpipe tobacco smoking: A systematic review
Source: PLoS One. 2018 Feb 9;13(2):e0192191. doi: 10.1371/journal.pone.0192191 (PMC5806869; doi:10.1371/journal.pone.0192191)
Supplement: S1 File — (DOCX) [file pone.0192191.s001.docx]

**Search strategies, prevalence definitions, and data from individual studies**

**Table A. Electronic search strategies**

| **Database** | **Search strategy** |
| --- | --- |
| MEDLINE (1950 onward) | Waterpipe*.mp.  “water pipe*”.mp.  shisha*.mp.  sheesha*.mp.  hooka*.mp.  huqqa*.mp.  guza*.mp.  goza*.mp.  narghil*.mp.  nargil*.mp.  arghil*.mp  argil*.mp  (hubbl* adj3 bubbl*).mp.  or/1-13 |
| EMBASE (1988 onward) | Waterpipe*.mp.  “water pipe*”.mp.  shisha*.mp.  sheesha*.mp.  hooka*.mp.  huqqa*.mp.  guza*.mp.  goza*.mp.  narghil*.mp.  nargil*.mp.  arghil*.mp  argil*.mp  (hubbl* adj3 bubbl*).mp.  or/1-13 |
| ISI the Web of Science | (waterpipe* OR "water pipe*" OR shisha* OR sheesha* OR hooka* OR huqqa* OR guza* OR goza* OR narghil* OR nargil* OR argil* OR arghil* OR (hubbl* SAME bubbl*)) |

**Table B. Categorisation of prevalence measures**

| **Category** | **Examples** |
| --- | --- |
| Past 30 day use | N/A – only studies reporting past 30 day use were included in this category |
| Ever use | Ever  Lifetime |
| Regular or occasional use | At least one waterpipe weekly and smoking regularly  Daily or less than daily  Daily or occasional  Daily or occasional AND weekly  Daily or some days  Less than monthly, less than weekly, weekly, or daily  Less than weekly, weekly, or daily  Once in lifetime and now smoking every day or some days  Regular or occasional |
| Daily use | Daily  Near daily |
| Other use | At least 12 times in the last year  At least 20 times in lifetime  At least monthly  At least monthly for the last year  At least six times in the past year  At least weekly  At least weekly for the last 6 months  Ever on a daily basis  Ever regular  More than 9 times  Occasional  Once in lifetime and now smoking every day , some days, or rarely  Past 3 months  Past week  Past year  Rarely, some days, or every day  Smoking at time of study  Three or more times per week |
| Unspecified use | Current  Present  Regular  Not reported |

**Table C. Past 30 day waterpipe tobacco use among youth**

| **Author** | **Country** | **Year** | **Mean age (SD); age range** | **Level of jurisdiction, location** | **Name of survey tool** | **Newcastle-Ottawa Scale*** | **Past 30 day prevalence (95% CI)** |
| --- | --- | --- | --- | --- | --- | --- | --- |
| **Eastern Mediterranean Region** | | | | | | | |
| Jawad 2015 | Djibouti | 2009 | 13 to 15 | National, School | Global Youth Tobacco Survey | 4/2 | 7.1 (5.7-8.5) |
| El Awa 2013^ | Egypt | 2009 | 13 to 15 | National, School | Global Youth Tobacco Survey | 5/2 | 3.4 (2.9-3.9) |
| Jawad 2015 | Egypt | 2009 | 13 to 15 | National, School | Global Youth Tobacco Survey | 4/2 | 8.5 (7.7-9.3) |
| Baheiraei 2013 | Iran | 2010 | Not reported | City, Household | Not reported | 1/2 | 28.0 (25.5-30.5) |
| Keshavarz 2013 | Iran | 2010 | 19+ | National, University | Global Health Professional Students Survey | 4/2 | 15.7 (11.7-19.6) |
| Ziaei 2016 | Iran | 2013 | 16.1; 15 to 17 | City, School | Global Youth Tobacco Survey | 5/2 | 9.7 (8.2-11.2) |
| CDC 2009 | Iraq | 2008 | 13 to 15 | City, School | Global Youth Tobacco Survey | 5/2 | 6.3 (5.3-7.3) |
| Hussain 2013 | Iraq | 2012 | 13 to 18 | City, School | Global Youth Tobacco Survey | 4/2 | 4.8 (3.8-5.8) |
| Khader 2009 | Gaza Strip | 2008 | <12 to >16 | National, School | Global Youth Tobacco Survey | 5/2 | 16.0 (14.1-17.9) |
| Alzyoud 2012 | Jordan | 2012 | 14.7 (1.9); 11 to 17 | Subnational, School | Arabic Youth Tobacco Use Composite Measure | 4/2 | 33.6 (30.7-36.6) |
| Jawad 2015 | Jordan | 2009 | 13 to 15 | National, School | Global Youth Tobacco Survey | 4/2 | 18.9 (17.1-20.7) |
| Khader 2009 | Jordan | 2008 | <12 to >16 | National, School | Global Youth Tobacco Survey | 5/2 | 22.1 (20.1-24.1) |
| McKelvey 2013 | Jordan | 2008 | Not reported | Subnational, School | Not reported | 3/2 | 14.0 (12.4-15.6) |
| McKelvey 2013 | Jordan | 2009 | 13.3 (0.7) | Subnational, School | Not reported | 3/2 | 12.4 (10.9-13.8) |
| McKelvey 2013 | Jordan | 2010 | 14.7 (0.6) | Subnational, School | Not reported | 3/2 | 15.3 (13.8-16.9) |
| McKelvey 2013 | Jordan | 2011 | 15.7 (0.6) | Subnational, School | Not reported | 3/2 | 22.6 (20.8-24.5) |
| Jawad 2015 | Kuwait | 2009 | 13 to 15 | National, School | Global Youth Tobacco Survey | 4/2 | 14.8 (13.5-16.1) |
| El-Roueiheb 2008 | Lebanon | 2002 | 15.0 (1.8) | City, School | Not reported | 1/2 | 29.6 (27.8-31.4) |
| Jawad 2015 | Lebanon | 2011 | 13 to 15 | National, School | Global Youth Tobacco Survey | 4/2 | 36.9 (34.9-38.9) |
| Jawad 2015 B | Lebanon | 2011 | 12.3 (1.2); <12 to >13 | National, School | Not reported | 4/2 | 22.1 (19.7-24.5) |
| Jradi 2013 | Lebanon | 2009 | 23.6 (1.0); 21 to 26 | National, University | Global Health Professional Students Survey | 4/2 | 29.5 (23.0-36.0) |
| Khader 2009 | Lebanon | 2008 | <12 to >16 | National, School | Global Youth Tobacco Survey | 4/2 | 37.2 (34.9-39.5) |
| Saade 2008 | Lebanon | 2005 | 13 to 15 | National, School | Global Youth Tobacco Survey | 4/2 | 33.9 (32.3-35.5) |
| Saade 2008 | Lebanon | 2001 | 13 to 15 | National, School | Global Youth Tobacco Survey | 4/2 | 33.4 (32.1-34.7) |
| Jawad 2015 | Libya | 2010 | 13 to 15 | National, School | Global Youth Tobacco Survey | 4/2 | 4.6 (3.7-5.5) |
| Jawad 2015 | Morocco | 2010 | 13 to 15 | National, School | Global Youth Tobacco Survey | 4/2 | 6.8 (6.0-7.6) |
| Al-Lawati 2008 | Oman | 2003 | 15.0 (1.5); <13 to 17+ | Subnational, School | Global Youth Tobacco Survey | 5/2 | 9.6 (8.3-10.9) |
| Jawad 2015 | Oman | 2010 | 13 to 15 | National, School | Global Youth Tobacco Survey | 4/2 | 2.5 (1.7-3.3) |
| Al Moamary 2012 | Saudi Arabia | 2010 | 16 to 18 | City, School | Global Youth Tobacco Survey | 5/2 | 10.2 (8.5-11.9) |
| Jawad 2015 | Saudi Arabia | 2010 | 13 to 15 | National, School | Global Youth Tobacco Survey | 4/2 | 10.2 (9.0-11.4) |
| Jawad 2015 | Sudan | 2009 | 13 to 15 | National, School | Global Youth Tobacco Survey | 4/2 | 5.5 (4.4-6.6) |
| Jawad 2015 | Syria | 2010 | 13 to 15 | National, School | Global Youth Tobacco Survey | 4/2 | 20.1 (18.1-22.1) |
| Khader 2009 | Syria | 2008 | <12 to >16 | National, School | Global Youth Tobacco Survey | 4/2 | 32.3 (30.1-34.5) |
| Harrabi 2010 | Tunisia | 2003 | 15.0 (1.5); 13 to 17 | Subnational, School | Not reported | 2/2 | 5.2 (4.1-6.3) |
| Jawad 2015 | Tunisia | 2010 | 13 to 15 | National, School | Global Youth Tobacco Survey | 4/2 | 6.7 (5.5-7.9) |
| Jawad 2015 | United Arab Emirates | 2005 | 13 to 15 | National, School | Global Youth Tobacco Survey | 4/2 | 9.5 (9.0-10.0) |
| Jawad 2015 | West Bank | 2009 | 13 to 15 | National, School | Global Youth Tobacco Survey | 4/2 | 32.7 (30.6-34.8) |
| Khader 2009 | West Bank | 2008 | <12 to >16 | National, School | Global Youth Tobacco Survey | 4/2 | 33.5 (31.3-35.7) |
| Jawad 2015 | Yemen | 2008 | 13 to 15 | National, School | Global Youth Tobacco Survey | 4/2 | 2.7 (1.6-3.8) |
| **European Region** | | | | | | | |
| Jawad 2015 | Azerbaijan | 2011 | 13 to 15 | National, School | Global Youth Tobacco Survey | 4/2 | 3.8 (3.0-4.6) |
| Jawad 2015 | Czech Republic | 2011 | 13 to 15 | National, School | Global Youth Tobacco Survey | 4/2 | 22.1 (20.7-23.5) |
| Jawad 2015 | Estonia | 2007 | 13 to 15 | National, School | Global Youth Tobacco Survey | 4/2 | 21.9 (20.4-23.4) |
| Kuntz 2015 | Germany | 2009 | 12 to 17 | National, Household | German Health Interview and Examination Survey | 2/2 | 10.0 (9.1-10.9) |
| Jawad 2015 | Hungary | 2008 | 13 to 15 | National, School | Global Youth Tobacco Survey | 4/2 | 12.1 (11.0-13.2) |
| Jawad 2015 | Latvia | 2011 | 13 to 15 | National, School | Global Youth Tobacco Survey | 4/2 | 22.7 (21.3-24.1) |
| Jawad 2015 | Poland | 2009 | 13 to 15 | National, School | Global Youth Tobacco Survey | 4/2 | 10.7 (9.6-11.8) |
| Jawad 2015 | Romania | 2009 | 13 to 15 | National, School | Global Youth Tobacco Survey | 4/2 | 2.2 (1.7-2.7) |
| Jawad 2015 | Slovakia | 2011 | 13 to 15 | National, School | Global Youth Tobacco Survey | 4/2 | 12.6 (11.6-13.6) |
| Jawad 2015 | Slovenia | 2011 | 13 to 15 | National, School | Global Youth Tobacco Survey | 4/2 | 8.3 (7.0-9.6) |
| Koprivnikar 2016 | Slovenia | 2014 | 15 years | National, School | Health Behavior in School-aged Children (HBSC) Survey | 2/2 | 3.0 (2.2-3.8) |
| Jawad 2015 | Ukraine | 2011 | 13 to 15 | National, School | Global Youth Tobacco Survey | 4/2 | 12.8 (11.7-13.9) |
| **Region of the Americas** | | | | | | | |
| Szklo 2011 | Brazil | 2009 | 13 to 15 | Subnational, School | Global Youth Tobacco Survey | 2/2 | 9.1 (8.1-10.0) |
| Chan 2011 | Canada | 2006 | Not reported | National, School | Canadian Youth Smoking Survey | 2/2 | 2.7 (2.5-2.9) |
| Cole 2014 | Canada | 2010 | Not reported | National, School | Canadian Youth Smoking Survey | 2/2 | 2.5 (2.2-2.7) |
| Czoli 2013 | Canada | 2010 | Not reported | National, School | Canadian Youth Smoking Survey | 3/2 | 4.0 (3.8-4.2) |
| Minaker 2015 | Canada | 2010 | 14 to 18 | National, School | Canadian Youth Smoking Survey | 2/2 | 4.0 (3.8-4.2) |
| Minaker 2015 | Canada | 2012 | 14 to 18 | National, School | Canadian Youth Smoking Survey | 3/2 | 5.4 (5.1-5.7) |
| Ambrose 2015 | United States | 2013 | 14.5 (0.0); 12 to 17 | National, Household | Population Assessment of Tobacco and Health (PATH) | 3/2 | 1.7 (1.5-1.9) |
| Amrock 2014 | United States | 2011 | <13 to 17+ | National, School | National Youth Tobacco Survey | 3/2 | 2.6 (2.4-2.8) |
| Arrazola 2014 | United States | 2013 | Not reported | National, School | National Youth Tobacco Survey | 3/2 | 1.1 (0.8-1.5) |
| Arrazola 2014 | United States | 2013 | Not reported | National, School | National Youth Tobacco Survey | 3/2 | 5.2 (4.6-6.0) |
| Arrazola 2014 | United States | 2014 | Not reported | National, School | National Youth Tobacco Survey | 3/2 | 2.5 (2.3-2.7) |
| Arrazola 2014 | United States | 2014 | Not reported | National, School | National Youth Tobacco Survey | 3/2 | 9.4 (9.0-9.8) |
| Barnett 2014 | United States | 2009 | Not reported | Subnational, School | Florida Youth Tobacco Survey | 3/2 | 7.7 (7.0-8.4) |
| Barnett 2014 | United States | 2010 | Not reported | Subnational, School | Florida Youth Tobacco Survey | 3/2 | 8.2 (7.9-8.5) |
| Barnett 2014 | United States | 2011 | Not reported | Subnational, School | Florida Youth Tobacco Survey | 3/2 | 8.0 (7.3-8.7) |
| Barnett 2014 | United States | 2012 | Not reported | Subnational, School | Florida Youth Tobacco Survey | 3/2 | 7.7 (7.4-8.0) |
| Blosnich 2011 | United States | 2008 | 20.2 (1.6); 18 to 24 | National, College | National College Health Assessment | 1/2 | 9.1 (8.9-9.3) |
| Bover Manderski 2012 | United States | 2008 | Not reported | Subnational, School | New Jersey Youth Tobacco Survey | 3/2 | 9.7 (8.6-10.8) |
| Bover Manderski 2012 | United States | 2010 | Not reported | Subnational, School | New Jersey Youth Tobacco Survey | 3/2 | 11.4 (10.2-12.6) |
| CDC 2013 | United States | 2011 | Not reported | National, School | National Youth Tobacco Survey | 3/2 | 4.1 (3.4-5.0) |
| CDC 2013 | United States | 2011 | Not reported | National, School | National Youth Tobacco Survey | 3/2 | 1.0 (0.8-1.4) |
| CDC 2013 | United States | 2012 | Not reported | National, School | National Youth Tobacco Survey | 3/2 | 5.4 (4.6-6.3) |
| CDC 2013 | United States | 2012 | Not reported | National, School | National Youth Tobacco Survey | 3/2 | 1.3 (1.0-1.7) |
| Cohn 2015 | United States | 2013 | 20.9; 18 to 24 | National, Household | Truth Initiative Young Adult Cohort Study | 2/2 | 4.0 (3.1-4.9) |
| Cohn 2016 | United States | 2011 | 21.0; 18 to 24 | National, Household | Truth Initiative Young Adult Cohort Study | 1/2 | 2.8 (2.3-3.3) |
| Cohn 2016 | United States | 2011 | 18 to 24 | National, Household | Truth Initiative Young Adult Cohort Study | 1/1 | 1.8 |
| Cohn 2016 | United States | 2012 | 18 to 24 | National, Household | Truth Initiative Young Adult Cohort Study | 1/1 | 3.5 |
| Cohn 2016 | United States | 2013 | 18 to 24 | National, Household | Truth Initiative Young Adult Cohort Study | 1/1 | 3.5 |
| Cohn 2016 | United States | 2014 | 18 to 24 | National, Household | Truth Initiative Young Adult Cohort Study | 1/1 | 3.8 |
| Cohn 2016 | United States | 2015 | 18 to 24 | National, Household | Truth Initiative Young Adult Cohort Study | 1/1 | 1.6 |
| Gilreath 2016 | United States | 2014 | Not reported | Subnational, School | Southern California Children's Health Study | 2/2 | 10.7 (9.4-12.0) |
| Jarrett 2012 | United States | 2008 | 18 to 24 | National, College | National College Health Assessment II | 3/2 | 9.6 (9.4-9.8) |
| Jordan 2010 | United States | 2008 | Not reported | Subnational, School | New Jersey Youth Tobacco Survey | 3/2 | 9.7 (8.6-10.8) |
| Primack 2009 | United States | 2005 | Median: 14 | Subnational, School | Arizona Youth Tobacco Survey | 4/2 | 3.5 (3.1-3.9) |
| Primack 2010 | United States | 2008 | 18+ | National, College | National College Health Assessment II | 4/2 | 7.2 (6.7-7.7) |
| Primack 2013 | United States | 2008 | 22.1 (5.5); 18 to 60 | National, College | National College Health Assessment | 2/2 | 8.4 (8.2-8.6) |
| Salloum 2016 | United States | 2013 | 18 to 24 | National, Household | Population Assessment of Tobacco and Health (PATH) | 3/2 | 11.0 (10.4-11.6) |
| Sidani 2013 | United States | 2008 | 18 to 25 | National, University | National College Health Assessment | 2/2 | 9.9 (9.7-10.1) |
| Singh 2016 | United States | 2015 | Not reported | National, School | National Youth Tobacco Survey | 1/2 | 2.0 (1.5-2.6) |
| Singh 2016 | United States | 2015 | Not reported | National, School | National Youth Tobacco Survey | 2/2 | 7.2 (6.3-8.3) |
| Villanti 2015 | United States | 2013 | 18 to 24 | National, Household | Legacy Young Adult Cohort Study | 2/2 | 4.0 (3.0-5.0) |
| **Western Pacific Region** | | | | | | | |
| Jiang 2016 | Hong Kong | 2012 | 14.8 (1.9); Not reported | National, School | School-based Survey on Smoking among Students | 3/2 | 1.2 (1.1-1.3) |

*Total score for the selection domain (out of five)/total score for the outcome domain (out of three); ^among females only

**Table D.** Ever waterpipe tobacco use among adults

| **Author and year** | **Country** | **Year** | **Mean age (SD); age range** | **Level of jurisdiction, Location** | **Name of survey tool** | **Newcastle-Ottawa Scale*** | **Ever prevalence (95% CI)** |
| --- | --- | --- | --- | --- | --- | --- | --- |
| **African Region** | | | | | | | |
| Kruger 2016 | South Africa | 2013 | Not reported | Subnational, University | Not reported | 2/2 | 63.0 (61.6-64.4) |
| **Region of the Americas** | | | | | | | |
| Agaku 2014b | United States | 2012 | 18+ | National, Household | Not reported | 2/2 | 5.0 (4.8-5.2) |
| Cavaros-Rehg 2015 | United States | 2013 | 18+ | National, Household | Truth Initiative Young Adult Cohort Study | 1/2 | 15.7 (15.2-16.2) |
| Grinberg 2015 | United States | 2010 | 18 to 30 | National, Household | Tobacco Use Supplement of the Current Population Survey | 2/2 | 5.3 (4.0-6.6) |
| Grinberg 2016 | United States | 2010 | 18 to 85 | National, Household | Tobacco Use Supplement of the Current Population Survey | 2/2 | 2.1 (2.1-2.2) |
| Grinberg 2016 | United States | 2010 | 18 to 40 | National, Household | Tobacco Use Supplement of the Current Population Survey | 2/2 | 3.9 (3.8-4.0) |
| McMillen 2012 | United States | 2010 | 18+ | National, Household | Social Climate Survey of Tobacco Control (SCS-TC) | 2/2 | 8.8 (7.8-9.8) |
| Park 2016 | United States | 2012 | 18+ | National, Household | National Adult Tobacco Survey | 3/2 | 12.3 (12.0-12.6) |
| Rath 2012 | United States | 2011 | 18 to 34 | National, Household | Legacy Young Adult Cohort Study | 2/2 | 17.0 (15.9-18.1) |
| Richardson 2014 | United States | 2011 | 18 to 34 | National, Household | Not reported | 1/2 | 15.6 (14.0-17.1) |
| Richardson 2014 | United States | 2012 | 18 to 34 | National, Household | Not reported | 1/2 | 20.0 (18.3-21.7) |
| Richardson 2014 | United States | 2012 | 18 to 34 | National, Household | Not reported | 1/2 | 21.8 (20.0-23.5) |
| Salloum 2015 | United States | 2009 | 18+ | National, Household | National Adult Tobacco Survey | 3/2 | 9.8 (9.6-10.0) |
| Smith 2011^ | United States | 2005 | 18+ | Subnational, Household | California Tobacco Survey (CTS) | 2/2 | 7.9 (7.5-8.3) |
| Smith 2011^$^ | United States | 2005 | 18+ | Subnational, Household | California Tobacco Survey (CTS) | 2/2 | 1.9 (1.7-2.1) |
| Smith 2011^ | United States | 2008 | 18+ | Subnational, Household | California Tobacco Survey (CTS) | 2/2 | 11.2 (10.6-11.8) |
| Smith 2011^$^ | United States | 2008 | 18+ | Subnational, Household | California Tobacco Survey (CTS) | 2/2 | 2.8 (2.5-3.1) |
| Zhang 2016 | United States | 2010 | 18+ | National, Household | Tobacco Use Supplement of the Current Population Survey | 1/2 | 2.7 (2.6-2.8) |
| **Eastern Mediterranean Region** | | | | | | | |
| Khami 2010 | Iran | 2005 | 26.0; 21 to 42 | National, University | Not reported | 2/2 | 20.9 (16.0-25.8) |
| Sarrafzadegan 2010 | Iran | 2009 | 41.2 (16.0); 19+ | Subnational, Household | Isfahan Healthy Heart Program | 3/2 | 41.4 (40.5-42.3) |
| Ziaaddini 2013 | Iran | 2012 | 12+ | City, Household | Not reported | 2/2 | 35.7 (32.6-38.8) |
| Azab 2013^$^ | Jordan | 2011 | 17+ | National, Maternity clinics | Not reported | 2/2 | 35.6 (31.4-39.8) |
| Saade 2009 | Lebanon | 2005 | Not reported | National, University | Global Health Professional Students Survey | 2/2 | 65.3 (62.0-68.5) |
| Ward 2015 | Syria | 2004 | 40.8 (10.5); 18+ | City, Household | Aleppo Household Surveys | 2/2 | 15.8 (14.4-17.2) |
| **European Region** | | | | | | | |
| Baron-Epel 2015 | Israel | 2012 | 45.4; 30 to 65 | Subnational, Household | Not reported | 2/2 | 21.8 (19.1-24.5) |
| Grant 2014 | United Kingdom | 2012 | 18+ | National, Household | Not reported | 1/2 | 11.6 (11.2-12.0) |

*Total score for the selection domain (out of five)/total score for the outcome domain (out of three); ^Among males only; ^$^Among females only

**Table E. Ever waterpipe tobacco use among youth**

| **Author and year** | **Country** | **Year** | **Mean age (SD); Age range** | **Level of jurisdiction, Location** | **Name of survey tool** | Newcastle-Ottawa Scale* | Ever prevalence (95% CI) |
| --- | --- | --- | --- | --- | --- | --- | --- |
| **Region of the Americas** | | | | | | | |
| Reveles 2013 | Brazil | 2011 | 10 to 19 years | Subnational, School | Not reported | 2/2 | 19.7 (16.2-23.2) |
| Chan 2011 | Canada | 2006 | Not reported | National, School | Canadian Youth Smoking Survey | 2/2 | 6.8 (6.6-7.0) |
| Czoli 2013 | Canada | 2010 | Not reported | National, School | Canadian Youth Smoking Survey | 3/2 | 10.1 (9.8-10.4) |
| Minaker 2015 | Canada | 2012 | 14 to 18 | National, School | Canadian Youth Smoking Survey | 3/2 | 14.3 (13.9-14.7) |
| Ambrose 2015 | United States | 2013 | 14.5 (0.0); 12 to 17 | National, Household | Population Assessment of Tobacco and Health (PATH) | 3/2 | 7.4 (7.0-7.8) |
| Amrock 2014 | United States | 2011 | <13 to 17+ | National, School | National Youth Tobacco Survey | 3/2 | 7.3 (6.9-7.7) |
| Arrazola 2014a | United States | 2013 | Not reported | National, School | National Youth Tobacco Survey | 3/2 | 3.0 (2.4-3.7) |
| Arrazola 2014a | United States | 2013 | Not reported | National, School | National Youth Tobacco Survey | 3/2 | 14.3 (12.-7-16.0) |
| Barnett 2009 | United States | 2007 | Not reported | Subnational, School | Florida Youth Tobacco Survey | 3/2 | 6.8 (6.3-7.3) |
| Barnett 2014 | United States | 2007 | Not reported | Subnational, School | Florida Youth Tobacco Survey | 3/2 | 10.7 (9.7-11.7) |
| Barnett 2014 | United States | 2008 | Not reported | Subnational, School | Florida Youth Tobacco Survey | 3/2 | 13.5 (13.2-13.8) |
| Barnett 2014 | United States | 2009 | Not reported | Subnational, School | Florida Youth Tobacco Survey | 3/2 | 15.8 (14.9-16.7) |
| Barnett 2014 | United States | 2010 | Not reported | Subnational, School | Florida Youth Tobacco Survey | 3/2 | 16.6 (16.2-17.0) |
| Barnett 2014 | United States | 2011 | Not reported | Subnational, School | Florida Youth Tobacco Survey | 3/2 | 18.2 (17.2-19.2) |
| Barnett 2014 | United States | 2012 | Not reported | Subnational, School | Florida Youth Tobacco Survey | 3/2 | 16.7 (16.3-17.1) |
| Bover Manderski 2012 | United States | 2008 | Not reported | Subnational, School | New Jersey Youth Tobacco Survey | 3/2 | 17.9 (16.5-19.3) |
| Bover Manderski 2012 | United States | 2010 | Not reported | Subnational, School | New Jersey Youth Tobacco Survey | 3/2 | 20.9 (19.3-22.5) |
| Cohn 2015 | United States | 2013 | 20.9 (2.0); 18 to 24 | National, Household | Truth Initiative Young Adult Cohort Study | 2/2 | 17.0 (15.3-18.7) |
| Gilreath 2016 | United States | 2014 | Not reported | Subnational, School | Southern California Children's Health Study | 2/2 | 16.5 (14.9-18.1) |
| Jarrett 2012 | United States | 2008 | 18 to 24 | National, College | National College Health Assessment II | 3/2 | 32.5 (32.2-32.8) |
| Primack 2009 | United States | 2005 | Median: 14 | Subnational, School | Arizona Youth Tobacco Survey | 4/2 | 6.4 (5.8-7.0) |
| Primack 2010 | United States | 2008 | 18+ | National, College | National College Health Assessment II | 4/2 | 29.5 (28.5-30.5) |
| Primack 2013 | United States | 2008 | 22.1 (5.5); 18 to 60 | National, College | National College Health Assessment | 2/2 | 30.5 (30.2-30.8) |
| Salloum 2016 | United States | 2013 | 18 to 24 | National, Household | Population Assessment of Tobacco and Health (PATH) | 3/2 | 44.0 (43.0-45.0) |
| Sidani 2013 | United States | 2008 | 18 to 25 | National, University | National College Health Assessment | 2/2 | 32.7 (32.4-33.0) |
| Villanti 2015b | United States | 2013 | 18 to 24 | National, Household | Legacy Young Adult Cohort Study | 2/2 | 23.0 (20.9-25.1) |
| **Eastern Mediterranean Region** | | | | | | | |
| El-Gilany 2008 | Egypt | 2005 | 15.9 (1.1); 14 to 19 | Subnational, School | Global Youth Tobacco Survey | 4/2 | 14.9 (12.9-16.9) |
| Khader 2009 | Gaza Strip | 2008 | <12 to >16 | National, School | Global Youth Tobacco Survey | 5/2 | 25.8 (23.6-28.0) |
| Baheiraei 2013 | Iran | 2010 | Not reported | City, Household | Not reported | 1/2 | 45.1 (42.3-47.9) |
| Fakhari 2015 | Iran | 2010 | 15.7 (0.7); 14 to 19 | Subnational, School | Not reported | 3/2 | 48.9 (46.5-50.3) |
| Keshavarz 2013 | Iran | 2010 | 19+ | National, University | Global Health Professional Students Survey | 4/2 | 50.8 (45.3-56.2) |
| Mohammadpoorasl 2014 | Iran | 2011 | 22.1; 18 to 34 | Subnational, University | Not reported | 1/2 | 39.4 (37.2-41.6) |
| Ramezankhani 2010 | Iran | 2008 | 14.7 (2.1) | City, School | Global Youth Tobacco Survey | 2/2 | 54.9 (53.4-56.4) |
| Roohafza 2015 | Iran | 2010 | 15.4 (1.7) | Subnational, School | Not reported | 3/2 | 32.3 (31.1-33.5) |
| Ziaaddini 2010 | Iran | 2009 | 17.9 (0.6) | City, School | Not reported | 2/2 | 51.5 (47.5-55.5) |
| Ziaei 2016 | Iran | 2013 | 16.1; 15 to 17 | City, School | Global Youth Tobacco Survey | 5/2 | 21.6 (19.5-23.7) |
| CDC 2009 | Iraq | 2008 | 13 to 15 | City, School | Global Youth Tobacco Survey | 5/2 | 12.9 (11.5-14.3) |
| Khader 2009 | Jordan | 2008 | <12 to >16 | National, School | Global Youth Tobacco Survey | 5/2 | 38.3 (36.0-40.6) |
| McKelvey 2013 | Jordan | 2008 | 12.7 (0.6) | Subnational, School | Not reported | 3/2 | 30.2 (28.1-32.3) |
| McKelvey 2013 | Jordan | 2009 | 13.3 (0.7) | Subnational, School | Not reported | 3/2 | 36.1 (34.0-38.2) |
| McKelvey 2013 | Jordan | 2010 | 14.7 (0.6) | Subnational, School | Not reported | 3/2 | 44.4 (42.2-46.5) |
| McKelvey 2013 | Jordan | 2011 | 15.7 (0.6) | Subnational, School | Not reported | 3/2 | 51.7 (49.5-53.9) |
| Bejjani 2012 | Lebanon | 2009 | 14.6 (1.1); 12 to 19 | National, School | European School Survey Project on Alcohol and Drugs | 3/2 | 42.4 (39.5-45.3) |
| El-Roueiheb 2008 | Lebanon | 2002 | 15.0 (1.8) | City, School | Not reported | 1/2 | 65.3 (63.4-67.2) |
| Jawad 2015b | Lebanon | 2011 | 12.3 (1.2); <12 to >13 | National, School | Not reported | 4/2 | 44.3 (41.4-47.2) |
| Jradi 2013 | Lebanon | 2009 | 23.6 (1.0); 21 to 26 | National, University | Global Health Professional Students Survey | 4/2 | 41.9 (34.9-48.9) |
| Khader 2009 | Lebanon | 2008 | <12 to >16 | National, School | Global Youth Tobacco Survey | 4/2 | 54.4 (52.0-56.8) |
| Saade 2008 | Lebanon | 2001 | 13 to 15 | National, School | Global Youth Tobacco Survey | 4/2 | 57.2 (55.8-58.6) |
| Saade 2008 | Lebanon | 2005 | 13 to 15 | National, School | Global Youth Tobacco Survey | 4/2 | 55.7 (54.0-57.4) |
| Al-Lawati 2008 | Oman | 2003 | 15.0 (1.5); <13 to 17+ | Subnational, School | Global Youth Tobacco Survey | 5/2 | 26.6 (24.6-28.6) |
| Al Moamary 2012 | Saudi Arabia | 2010 | 16 to 18 | City, School | Global Youth Tobacco Survey | 5/2 | 33.0 (30.4-35.6) |
| Al-Bedah 2012 | Saudi Arabia | 2010 | Not reported | National, University | Global Health Professional Students Survey | 2/2 | 33.0 (29.7-36.2) |
| Khader 2009 | Syria | 2008 | <12 to >16 | National, School | Global Youth Tobacco Survey | 4/2 | 49.7 (47.4-52.0) |
| Harrabi 2010 | Tunisia | 2003 | 15.0 (1.5); 13 to 17 | Subnational, School | Not reported | 2/2 | 19.3 (17.3-21.3) |
| Khader 2009 | West Bank | 2008 | <12 to >16 | National, School | Global Youth Tobacco Survey | 4/2 | 47.6 (45.2-50.0) |
| **European Region** | | | | | | | |
| Parna 2008 | Estonia | 2006 | 11 to 15 | National, School | Health Behavior in School-aged Children (HBSC) Survey | 1/2 | 20.7 (19.5-21.8) |
| Kinnunen 2015 | Finland | 2013 | 12 to 18 | National, Household | Adolescent Health and Lifestyle Survey | 2/2 | 15.3 (14.1-16.5) |
| Krause 2016 | Germany | 2010 | 11 to 17 | Subnational, Household | Not reported | 2/2 | 20.0 (18.1-21.9) |
| Kuntz 2015 | Germany | 2009 | 12 to 17 | National, Household | German Health Interview and Examination Survey | 2/2 | 28.9 (27.6-30.2) |
| Korn 2008 | Israel | 2002 | 11 to 15 | National, School | Health Behavior in School-aged Children (HBSC) Survey | 4/2 | 37.7 (36.5-38.9) |
| Nadasan 2016 | Romania | 2014 | 14.9 (0.5) | City, School | Not reported | 2/2 | 21.1 (19.2-23.0) |
| Galanti 2015 | Sweden | 2011 | 15 to 17 | National, School | Not reported | 2/2 | 49.5 (48.5-50.6) |
| Evren 2014 | Turkey | 2012 | 16.5 (2.7) | City, School | Not reported | 3/2 | 45.8 (44.4-47.2) |
| Jawad 2013 | United Kingdom | 2011 | 14.5 (1.8); 15+ | Subnational, School | Not reported | 3/2 | 24.0 (22.3-25.7) |
| Jawad 2014b | United Kingdom | 2013 | Not reported | City, School | Young People's Lifestyle Survey | 3/2 | 12.0 (10.2-13.8) |
| Jawad 2016 | United Kingdom | 2014 | 13.7 (1.3); 11 to 17 | Subnational, School | Not reported | 1/2 | 40.1 (38.0-42.2) |

*Total score for the selection domain (out of five)/total score for the outcome domain (out of three);

**Table F. Regular or occasional waterpipe tobacco use among adults**

| **Author and year** | **Country** | **Year** | **Mean age (SD); Age range** | **Level of jurisdiction, Location** | **Name of survey tool** | **Newcastle-Ottawa Scale*** | **Regular or occasional prevalence (95% CI)** |
| --- | --- | --- | --- | --- | --- | --- | --- |
| **African Region** | | | | | | | |
| Agaku 2014a | Nigeria | 2012 | 15+ | National, Household | Global Adult Tobacco Survey | 2/2 | 0.3 (0.2-0.4) |
| **Region of the Americas** | | | | | | | |
| Agaku 2014a | Argentina | 2012 | 15+ | National, Household | Global Adult Tobacco Survey | 4/2 | 0.0 (0.0-0.0) |
| Agaku 2014a and Morton 2014 | Brazil | 2008 | 15+ | National, Household | Global Adult Tobacco Survey | 5/2 | 0.1 (0.1-0.1) |
| Menezes 2016 | Brazil | 2013 | 18 to 59 | National, Household | National Health Survey (PNS) | 3/2 | 1.2 (1.1-1.3) |
| Agaku 2014a and Morton 2014 | Mexico | 2009 | 15+ | National, Household | Global Adult Tobacco Survey | 5/2 | 0.0 (0.0-0.0) |
| Agaku 2014b | United States | 2012 | 18+ | National, Household | Not reported | 2/2 | 0.5 (0.4-0.6) |
| Sean Hu 2016 | United States | 2013 | 18+ | National, Household | National Adult Tobacco Survey | 3/2 | 0.6 (0.5-0.7) |
| Zhang 2016 | United States | 2010 | 18+ | National, Household | Tobacco Use Supplement of the Current Population Survey | 1/2 | 0.5 (0.5-0.5) |
| Agaku 2014a and Morton 2014 | Uruguay | 2009 | 15+ | National, Household | Global Adult Tobacco Survey | 5/2 | 0.1 (0.0-0.2) |
| **Eastern Mediterranean Region** | | | | | | | |
| Agaku 2014a, Jawad 2014a, and Morton 2014 | Egypt | 2009 | 15+ | National, Household | Global Adult Tobacco Survey | 5/2 | 3.3 (3.1-3.5) |
| El Awa 2013^ | Egypt | 2009 | 15+ | National, Household | Global Adult Tobacco Survey | 5/2 | 0.3 (0.2-0.4) |
| Khami 2010 | Iran | 2005 | 26.0; 21 to 42 | National, University | Not reported | 2/2 | 16.3 (11.9-20.8) |
| Mirahmadizadeh 2008 | Iran | 2004 | Not reported | Subnational, Health houses | WHO classification | 1/2 | 8.0 (7.0-9.0) |
| Ward 2015 | Syria | 2004 | 40.8 (10.5); 18+ | City, Household | Aleppo Household Surveys (AHS-1 and AHS-2) | 2/2 | 11.3 (10.1-12.5) |
| Aden 2013 | United Arab Emirates | 2011 | 29.0 (7.6); 18+ | Subnational, Not reported | Not reported | 1/2 | 4.8 (4.4-5.2) |
| **European Region** | | | | | | | |
| Agaku 2014a | Austria | 2012 | 15+ | National, Household | The Special Eurobarometer 385 (77.1) | 3/2 | 7.8 (6.2-9.4) |
| Agaku 2014a | Belgium | 2012 | 15+ | National, Household | The Special Eurobarometer 385 (77.1) | 3/2 | 4.7 (3.4-6.0) |
| Agaku 2014a | Bulgaria | 2012 | 15+ | National, Household | The Special Eurobarometer 385 (77.1) | 3/2 | 1.4 (0.7-2.1) |
| Agaku 2014a | Cyprus | 2012 | 15+ | National, Household | The Special Eurobarometer 385 (77.1) | 3/2 | 8.5 (6.1-10.9) |
| Agaku 2014a | Czech Republic | 2012 | 15+ | National, Household | The Special Eurobarometer 385 (77.1) | 3/2 | 7.9 (6.2-9.6) |
| Agaku 2014a | Denmark | 2012 | 15+ | National, Household | The Special Eurobarometer 385 (77.1) | 3/2 | 8.4 (6.7-10.1) |
| Agaku 2014a | Estonia | 2012 | 15+ | National, Household | The Special Eurobarometer 385 (77.1) | 3/2 | 7.6 (6.0-9.2) |
| Agaku 2014a | Finland | 2012 | 15+ | National, Household | The Special Eurobarometer 385 (77.1) | 3/2 | 1.5 (0.7-2.3) |
| Agaku 2014a | France | 2012 | 15+ | National, Household | The Special Eurobarometer 385 (77.1) | 3/2 | 7.1 (5.6-8.6) |
| Agaku 2014a | Germany | 2012 | 15+ | National, Household | The Special Eurobarometer 385 (77.1) | 3/2 | 5.0 (3.9-6.1) |
| Agaku 2014a | Greece | 2012 | 15+ | National, Household | The Special Eurobarometer 385 (77.1) | 3/2 | 2.9 (1.9-3.9) |
| Agaku 2014a | Hungary | 2012 | 15+ | National, Household | The Special Eurobarometer 385 (77.1) | 3/2 | 2.4 (1.5-3.3) |
| Agaku 2014a | Ireland | 2012 | 15+ | National, Household | The Special Eurobarometer 385 (77.1) | 3/2 | 0.9 (0.3-1.5) |
| Baron-Epel 2015 | Israel | 2012 | 45.4; 30 to 65 | Subnational, Household | Not reported | 2/2 | 12.8 (1.07-15.0) |
| Agaku 2014a | Italy | 2012 | 15+ | National, Household | The Special Eurobarometer 385 (77.1) | 3/2 | 2.0 (1.1-2.9) |
| Agaku 2014a | Latvia | 2012 | 15+ | National, Household | The Special Eurobarometer 385 (77.1) | 3/2 | 11.5 (9.5-13.5) |
| Agaku 2014a | Lithuania | 2012 | 15+ | National, Household | The Special Eurobarometer 385 (77.1) | 3/2 | 9.0 (7.2-10.8) |
| Agaku 2014a | Luxembourg | 2012 | 15+ | National, Household | The Special Eurobarometer 385 (77.1) | 3/2 | 7.8 (5.5-10.1) |
| Agaku 2014a | Malta | 2012 | 15+ | National, Household | The Special Eurobarometer 385 (77.1) | 3/2 | 2.8 (1.4-4.2) |
| Agaku 2014a | Netherlands | 2012 | 15+ | National, Household | The Special Eurobarometer 385 (77.1) | 3/2 | 5.1 (3.7-6.5) |
| Agaku 2014a | Portugal | 2012 | 15+ | National, Household | The Special Eurobarometer 385 (77.1) | 3/2 | 1.9 (1.1-2.7) |
| Agaku 2014a | Romania | 2011 | 15+ | National, Household | Global Adult Tobacco Survey | 4/2 | 0.4 (0.2-0.6) |
| Agaku 2014a and Morton 2014 | Russia | 2009 | 15+ | National, Household | Global Adult Tobacco Survey | 5/2 | 3.8 (3.4-4.2) |
| Jawad 2014a | Russia | 2009 | 15+ | National, Household | Global Adult Tobacco Survey | 5/2 | 2.7 (2.4-3.0) |
| Agaku 2014a | Slovakia | 2012 | 15+ | National, Household | The Special Eurobarometer 385 (77.1) | 3/2 | 4.2 (3.0-5.4) |
| Agaku 2014a | Slovenia | 2012 | 15+ | National, Household | The Special Eurobarometer 385 (77.1) | 3/2 | 2.0 (1.1-2.9) |
| Agaku 2014a | Spain | 2012 | 15+ | National, Household | The Special Eurobarometer 385 (77.1) | 3/2 | 2.9 (1.9-3.9) |
| Agaku 2014a | Sweden | 2012 | 15+ | National, Household | The Special Eurobarometer 385 (77.1) | 3/2 | 2.8 (1.8-3.8) |
| Agaku 2014a | Turkey | 2008 | 15+ | National, Household | Global Adult Tobacco Survey | 5/2 | 2.3 (2.0-2.6) |
| Erdol 2015 and Morton 2014 | Turkey | 2008 | 15+ | National, Household | Global Adult Tobacco Survey | 5/2 | 2.3 (2.0-2.6) |
| Erdol 2015 | Turkey | 2010 | 15+ | National, Household | Global Adult Tobacco Survey | 5/2 | 0.8 (0.6-1.0) |
| Agaku 2014a | Ukraine | 2010 | 15+ | National, Household | Global Adult Tobacco Survey | 5/2 | 2.1 (1.8-2.4) |
| Morton 2014 | Ukraine | 2010 | 15+ | National, Household | Global Adult Tobacco Survey | 5/2 | 2.0 (1.7-2.3) |
| Agaku 2014a | United Kingdom | 2012 | 15+ | National, Household | The Special Eurobarometer 385 (77.1) | 3/2 | 3.6 (2.6-4.6) |
| **South East Asia Region** | | | | | | | |
| Agaku 2014a and Morton 2014 | Bangladesh | 2009 | 15+ | National, Household | Global Adult Tobacco Survey | 5/2 | 0.7 (0.5-0.9) |
| Agaku 2014a | India | 2009 | 15+ | National, Household | Global Adult Tobacco Survey | 5/2 | 0.9 (0.8-1.0) |
| Jawad 2014a | India | 2010 | 15+ | National, Household | Global Adult Tobacco Survey | 5/2 | 0.8 (0.7-0.9) |
| Morton 2014 | India | 2010 | 15+ | National, Household | Global Adult Tobacco Survey | 5/2 | 0.9 (0.8-0.9) |
| Agaku 2014a | Indonesia | 2011 | 15+ | National, Household | Global Adult Tobacco Survey | 4/2 | 0.1 (0.0-0.2) |
| Agaku 2014a and Morton 2014 | Thailand | 2009 | 15+ | National, Household | Global Adult Tobacco Survey | 5/2 | 0.0 (0.0-0.0) |
| **Western Pacific Region** | | | | | | | |
| Agaku 2014a | China | 2010 | 15+ | National, Household | Global Adult Tobacco Survey | 5/2 | 0.4 (0.3-0.5) |
| Agaku 2014a | Malaysia | 2011 | 15+ | National, Household | Global Adult Tobacco Survey | 4/2 | 0.6 (0.4-0.8) |
| Agaku 2014a and Morton 2014 | Philippines | 2009 | 15+ | National, Household | Global Adult Tobacco Survey | 5/2 | 0.0 (0.0-0.0) |
| Agaku 2014a, Jawad 2014a, Morton 2014, and Xuan le 2013 | Vietnam | 2010 | 15+ | National, Household | Global Adult Tobacco Survey | 5/2 | 6.4 (5.9-6.9) |

*Total score for the selection domain (out of five)/total score for the outcome domain (out of three); ^Among females only

**Table G. ‘Other’ defined waterpipe tobacco use**

| **Author and year** | **Country** | **Year** | **Mean age (SD); Age range** | **Level of jurisdiction, Location** | **Name of survey tool** | **Newcastle-Ottawa Scale*** | **Waterpipe prevalence estimate (95% CI)** | **Waterpipe prevalence type (verbatim)** |
| --- | --- | --- | --- | --- | --- | --- | --- | --- |
| **African Region** | | | | | | | | |
| Khattab 2012 | Algeria | 2010 | 40+ | National, Household | Not reported | 2/2 | 0.5 (0.3-0.8) | Ever on a daily basis |
| **Region of the Americas** | | | | | | | | |
| Hamilton 2015 | Canada | 2013 | 16.0; <19 | Subnational, School | Ontario Student Drug Use and Health Survey | 5/2 | 12.5 (11.3-13.7) | Past-year |
| Larsen 2016 | Canada | 2013 | 15.9 (1.3); 12 to 19 | Subnational, School | Ontario Student Drug Use and Health Survey | 1/2 | 12.6 (11.4-13.8) | Past-year |
| Agaku 2014b | United States | 2012 | 18+ | National, Household | Not reported | 2/2 | 3.9 (3.7-4.1) | Once in lifetime and now smoking every day, some days, or rarely |
| Hampson 2013 | United States | 2013 | 20 to 21 | Subnational, Clinic | Oregon Youth Substance Use Project | 4/2 | 43.0 (39.3-46.7) | Past-year |
| Hampson 2013 | United States | 2013 | 20 to 21 | Subnational, Clinic | Oregon Youth Substance Use Project | 4/2 | 6.0 (4.2-7.8) | At least monthly in the last year |
| Hampson 2015 | United States | 2013 | 22.4; 20 to 25 | Subnational, Clinic | Oregon Youth Substance Use Project | 3/2 | 21.7 (18.9-24.5) | At least 20 times in lifetime |
| Palamar 2014 | United States | 2010 | Est 17-18 (Grade 12) | National, School | Monitoring the Future | 4/2 | 18.0 (17.0-19.0) | Past-year |
| Park 2016 | United States | 2012 | 18+ | National, Household | National Adult Tobacco Survey | 3/2 | 3.9 (3.7-4.1) | Rarely, some days or every day use |
| Primack 2015 | United States | 2010 | Est 17-18 (Grade 12) | National, School | Monitoring the Future | 4/2 | 7.2 (6.7-7.7) | At least six times in the past year |
| Primack 2015 | United States | 2010 | Est 17-18 (Grade 12) | National, School | Monitoring the Future | 4/2 | 18.8 (18.0-19.6) | Past-year |
| Primack 2015 | United States | 2010 | Est 17-18 (Grade 12) | National, School | Monitoring the Future | 3/2 | 17.1 (15.5-18.7) | Past-year |
| Primack 2015 | United States | 2011 | Est 17-18 (Grade 12) | National, School | Monitoring the Future | 3/2 | 18.5 (16.9-20.1) | Past-year |
| Primack 2015 | United States | 2012 | Est 17-18 (Grade 12) | National, School | Monitoring the Future | 3/2 | 18.3 (16.7-19.9) | Past-year |
| Primack 2015 | United States | 2013 | Est 17-18 (Grade 12) | National, School | Monitoring the Future | 3/2 | 21.4 (19.7-23.1) | Past-year |
| Sean Hu 2016 | United States | 2013 | 18+ | National, Household | National Adult Tobacco Survey | 3/2 | 4.3 (4.2-4.4) | Once in lifetime and now smoking every day, some days, or rarely |
| **Eastern Mediterranean Region** | | | | | | | | |
| Khattab 2012 | Egypt | 2010 | 40+ | National, Household | Not reported | 2/2 | 5.0 (4.5-5.4) | Ever on a daily basis |
| Fakhari 2015 | Iran | 2010 | 15.7 (0.7); 14 to 19 | Subnational, School | Not reported | 3/2 | 5.3 (4.7-5.9) | At least monthly |
| Kelishadi 2016 | Iran | 2011 | 12.5 (3.4); 6 to 18 | National, School | CASPIAN-IV Study | 5/2 | 1.8 (1.6-2.0) | Smoking at time of study |
| Mirahmadizadeh 2008 | Iran | 2004 | Not reported | Subnational, Health houses | WHO classification | 1/2 | 4.8 (4.0-5.6) | Occasional |
| Mohammadpoorasl 2014 | Iran | 2011 | 22.1; 18 to 34 | Subnational, University | Not reported | 1/2 | 8.5 (7.2-9.8) | At least monthly |
| Mohammadpoorasl 2014 | Iran | 2011 | 22.1; 18 to 34 | Subnational, University | Not reported | 1/2 | 3.4 (2.6-4.2) | At least weekly |
| Roohafza 2015 | Iran | 2010 | 15.4 (1.7) | Subnational, School | Not reported | 3/2 | 11.6 (10.7-12.5) | At least weekly |
| Sadjadi 2014 | Iran | 2013 | 53.1 (9.9); 40+ | Subnational, Household | Not reported | 1/2 | 8.0 (6.3-9.7) | At least weekly for the last 6 months |
| Sarrafzadegan 2010 | Iran | 2009 | 41.2 (16.0); 19+ | Subnational, Household | Isfahan Healthy Heart Program | 3/2 | 1.0 (0.8-1.2) | At least monthly |
| Zivari-Rahman 2012 | Iran | 2011 | Not reported | Subnational, University | Not reported | 2/2 | 38.3 (34.2-42.5) | At least weekly |
| Abu-Helalah 2015 | Jordan | 2014 | 33.9 (13.3); 18 to 79 | Subnational, Not reported | Global Adult Tobacco Survey | 4/2 | 6.8 (5.1-8.4) | Three or more times per week |
| Alzyoud 2012 | Jordan | 2012 | 14.7 (1.9); 11 to 17 | Subnational, School | Arabic Youth Tobacco Use Composite Measure | 4/2 | 30.1 (27.3-33.0) | Past-week |
| Alzyoud 2012 | Jordan | 2012 | 14.7 (1.9); 11 to 17 | Subnational, School | Arabic Youth Tobacco Use Composite Measure | 4/2 | 36.0 (33.0-38.9) | Past-year |
| Khattab 2012 | Jordan | 2010 | 40+ | National, Household | Not reported | 2/2 | 3.8 (3.1-4.4) | Ever on a daily basis |
| Bejjani 2012 | Lebanon | 2008 | 14.6 (1.1); 12 to 19 | National, School | European School Survey Project on Alcohol and Drugs | 3/2 | 18.9 (16.6-21.2) | More than 9 times |
| Khattab 2012 | Lebanon | 2010 | 40+ | National, Household | Not reported | 2/2 | 7.5 (6.6-8.4) | Ever on a daily basis |
| Salameh 2012 | Lebanon | 2009 | 40+ | National, Household | Not reported | 2/2 | 16.3 (14.8-17.9) | Ever regular |
| Khattab 2012 | Morocco | 2010 | 40+ | National, Household | Not reported | 2/2 | 0.3 (0.1-0.4) | Ever on a daily basis |
| Khattab 2012 | Pakistan | 2010 | 40+ | National, Household | Not reported | 2/2 | 2.2 (1.7-2.7) | Ever on a daily basis |
| Amin 2012 | Saudi Arabia | 2008 | 17.5 (1.0); 15 to 19 | Subnational, School | Global Youth Tobacco Survey | 4/2 | 26.9 (24.7-29.0) | Past-year |
| Khattab 2012 | Saudi Arabia | 2010 | 40+ | National, Household | Not reported | 2/2 | 10.0 (9.4-10.6) | Ever on a daily basis |
| Khattab 2012 | Syria | 2010 | 40+ | National, Household | Not reported | 2/2 | 2.3 (1.8-2.8) | Ever on a daily basis |
| Khattab 2012 | Tunisia | 2010 | 40+ | National, Household | Not reported | 2/2 | 1.4 (0.9-1.9) | Ever on a daily basis |
| Al-Houqani 2012 | United Arab Emirates | 2008 | 35.2 (13.8); 18+ | Subnational, Primary care clinic | Not reported | 2/2 | 0.8 (0.7-0.8) | Past-3 months |
| Khattab 2012 | United Arab Emirates | 2010 | 40+ | National, Household | Not reported | 2/2 | 3.7 (3.0-4.3) | Ever on a daily basis |
| **European Region** | | | | | | | | |
| Parna 2008 | Estonia | 2006 | 11 to 15 | National, School | Health Behavior in School-aged Children Survey | 1/2 | 3.4 (2.8-3.9) | At least weekly |
| Kuntz 2015 | Germany | 2009 | 12 to 17 | National, Household | German Health Interview and Examination Survey | 2/2 | 20.6 (19.4-21.8) | Past-year |
| Korn 2008 | Israel | 2002 | 11 to 15 | National, School | Health Behavior in School-aged Children Survey | 4/2 | 10.0 (9.3-10.7) | At least weekly |
| Galanti 2015 | Sweden | 2009 | 15 to 17 | National, School | Not reported | 1/2 | 7.0 (6.5-7.5) | At least 12 times in the last year |
| Galanti 2015 | Sweden | 2011 | 15 to 17 | National, School | Not reported | 2/2 | 9.9 (9.3-10.6) | At least monthly for the last year |
| Evren 2014 | Turkey | 2012 | 16.5 (2.7) | City, School | Not reported | 3/2 | 19.0 (17.9-20.1) | At least monthly |
| Khattab 2012 | Turkey | 2010 | 40+ | National, Household | Not reported | 2/2 | 0.5 (0.4-0.6) | Ever on a daily basis |
| Grant 2014 | United Kingdom | 2012 | 18+ | National, Household | Not reported | 1/2 | 1.0 (0.9-1.1) | At least monthly |

*Total score for the selection domain (out of five)/total score for the outcome domain (out of three)

**Table H. Unspecified definitions of waterpipe tobacco use**

| **Author and year** | **Country** | **Year** | **Mean age (SD); Age range** | **Level of jurisdiction, Location** | **Name of survey tool** | **Newcastle-Ottawa Scale*** | **Waterpipe prevalence estimate (95% CI)** |
| --- | --- | --- | --- | --- | --- | --- | --- |
| **Region of the Americas** | | | | | | | |
| Soneji 2015 | United States | 2010 | 15 to 23 | National, Household | Not reported | 2/2 | 20.1 (18.5-21.7) |
| **Eastern Mediterranean Region** | | | | | | | |
| Singh 2012 | Egypt | 2003 | 18+ | Subnational, Household | Not reported | 4/2 | 11.0 (10.1-11.9) |
| Abdollahifarid 2013 | Iran | 2010 | 39.0 (1.5); 18 to 73 | City, Household | Not reported | 3/2 | 11.9 (9.9-13.9) |
| Eslami 2013 | Iran | 2012 | 27.5 (6.5); <18 to 35+ | National, Hospital | Not reported | 5/2 | 6.2 (5.3-7.1) |
| Esmaielzadeh 2014 | Iran | 2011 | Not reported | Subnational, School | Youth Risk Behaviors Surveillance System Questionnaire | 3/2 | 59.0 (54.7-63.3) |
| Hamrah 2013 | Iran | 2009 | 50.9 (6.3); 40 to 64 | City, Household | Shahroud Eye Cohort Study | 3/2 | 0.7 (0.4-0.9) |
| Mohammad-Alizadeh 2015 | Iran | 2013 | 15.1 (1.0); 14 to 18 | Subnational, School | Not reported | 4/2 | 10.4 (8.9-11.9) |
| Tavafian 2009 | Iran | 2007 | 42.1 (16.5); 15+ | Subnational, Not reported | Not reported | 1/2 | 10.4 (8.9-11.9) |
| Salameh 2012 | Lebanon | 2009 | 40+ | National, Household | Not reported | 2/2 | 10.2 (8.9-11.4) |
| Basir 2014 | Pakistan | 2012 | 15.1 (1.3); 13 to 17 | City, School | Not reported | 1/2 | 39.0 (32.2-45.8) |
| Basir 2014 | Pakistan | 2012 | 25.1 (1.2); 23 to 27 | City, University | Not reported | 1/2 | 48.0 (41.1-54.9) |
| Khan 2015 | Pakistan | 2012 | 15 to 49 | National, Household | Demographic and Health Survey | 4/2 | 3.9 (3.5-4.2) |
| Al-Bedah 2012 | Saudi Arabia | 2010 | Not reported | National, University | Global Health Professional Students Survey | 2/2 | 16.6 (14.0-19.1) |
| Amin 2012 | Saudi Arabia | 2008 | 17.5 (1.0); 15 to 19 | Subnational, School | Global Youth Tobacco Survey | 4/2 | 11.7 (10.1-13.2) |
| **European Region** | | | | | | | |
| Kaluski 2009 | Israel | 2003 | 14.6; 11 to 19 | National, School | MABAT Youth Survey | 3/2 | 13.7 (12.9-14.6) |
| Baska 2008 | Latvia | 2007 | Not reported | National, School | Global Youth Tobacco Survey | 4/1 | 37.5 |
| Baska 2008 | Slovakia | 2007 | Not reported | National, School | Global Youth Tobacco Survey | 4/1 | 4.8 |
| **South East Asia Region** | | | | | | | |
| Khandelwal 2016 | India | 2015 | 13 to 17 | Subnational, School | Not reported | 1/2 | 6.4 (5.6-7.2) |

*Total score for the selection domain (out of five)/total score for the outcome domain (out of three)
